# Preferences for Alternative Care Modalities Among French Adults With Chronic Illness

**DOI:** 10.1001/jamanetworkopen.2021.41233

**Published:** 2021-12-29

**Authors:** Theodora Oikonomidi, Philippe Ravaud, Diana Barger, Viet-Thi Tran

**Affiliations:** 1 Université de Paris, Centre de Recherche Epidémiologie et StatistiqueS (CRESS), Institut National de la santé et de la recherche médicale (INSERM), Institut national de recherche pour l'agriculture, l'alimentation et l'environnement (INRAE), Paris, France; 2 Clinical Epidemiology Unit, Hôtel-Dieu Hospital, Assistance Publique-Hôpitaux de Paris, Paris, France; 3 Department of Epidemiology, Mailman School of Public Health, Columbia University, New York, New York; 4 University of Bordeaux, ISPED, Inserm Bordeaux Population Health, Team EMOS, UMR 1219, Bordeaux, France

## Abstract

**Importance:**

The COVID-19 pandemic led to the implementation of alternative care modalities (eg, teleconsultations and task shifting) that will continue to be implemented in parallel to traditional care after the pandemic. An ideal balance between alternative and traditional care modalities is unknown.

**Objectives:**

To quantify the ideal postpandemic balance between alternative and traditional care modalities among patients with chronic illness and to qualify the circumstances in which patients consider it appropriate to replace traditional care with alternative care.

**Design, Setting, and Participants:**

This survey study invited 5999 adults with chronic illness in ComPaRe, a French nationwide e-cohort of adults with chronic conditions who volunteer their time to participate in research projects, to participate in this study, which was performed from January 27 to February 23, 2021.

**Main Outcomes and Measures:**

Participants rated the ideal proportion at which they would use 3 alternative care modalities instead of the traditional care equivalent on a 0% to 100% scale (with 0% indicating using alternative care modalities for none of one's future care and 100% indicating using alternative care modalities for all of one's future care) of their overall future care: (1) teleconsultations, (2) online symptom-checkers to react to new symptoms, and (3) remote monitoring to adapt treatment outside consultations. The median ideal proportion of alternative care use was calculated. Perceived appropriate circumstances in which each alternative modality could replace traditional care were collected with open-ended questions. Analyses were performed on a weighted data set representative of patients with chronic illness in France.

**Results:**

Of the 5999 invited individuals, 1529 (mean [SD] age, 50.3 [14.7] years; 1072 [70.1%] female) agreed to participate (participation rate, 25.5%). Participants would choose teleconsultations for 50.0% of their future consultations (IQR, 11.0%-52.0%), online symptom-checkers over contacting their physician for 22.0% of new symptoms (IQR, 2.0%-50.0%), and remote monitoring instead of consultations for 52.3% of their treatment adaptations (IQR, 25.4%-85.4%). Participants reported 67 circumstances for which replacing traditional with alternative care modalities was considered appropriate, including 31 care activities (eg, prescription renewal and addressing acute or minor complaints), 25 patient characteristics (eg, stable chronic condition and established patient-physician relationship), and 11 required characteristics of the alternative care modalities (eg, quality assurance).

**Conclusions and Relevance:**

Results of this survey study suggest that after the pandemic, patients would choose alternative over traditional care for 22% to 52% of the time across different care needs. Participants proposed 67 criteria to guide clinicians in replacing traditional care with alternative care. These findings provide a guide for redesigning care in collaboration with patients after the pandemic.

## Key Points

**Question:**

What is an ideal balance between alternative care modalities implemented during the COVID-19 pandemic and traditional care in the postpandemic care model?

**Findings:**

This survey study of 1529 chronically ill adults found that patients would choose alternative care (ie, teleconsultations, symptom-checkers, and remote monitoring) over the traditional care equivalent for 22% to 52% of their future needs. The study identified 67 care activities, patient characteristics, and characteristics of alternative care modalities for which patients considered it appropriate to replace traditional with alternative care.

**Meaning:**

Alternative care modalities implemented during the pandemic could be used to deliver nearly half of patients’ postpandemic care.

## Teaser

This survey study of French patients with chronic illness performed after the start of the COVID-19 pandemic examined these patients’ beliefs on the ideal balance of alternative and traditional care modalities and the future of postpandemic care.

## Introduction

Half of adults in Western countries have at least 1 chronic condition, and 27% experience multimorbidity.^1^ The traditional care model fails to serve the increasing population of individuals with chronic illness because it is reactive and inflexible: physicians see patients most often only after they become ill, during in-person consultations scheduled at prespecified intervals.^2^ A more appropriate care model for patients with chronic illness would seek to prevent health deterioration, support patients outside consultations,^2^ and minimize treatment burden.^3,4^

The COVID-19 pandemic disrupted the traditional care model and forced physicians to implement alternative care modalities, ranging from technology-based remote care to organizational changes. For example, patients in France with COVID-19 were remotely monitored with daily self-reported questionnaires through the Covidom app,^5^ and separate hospital areas were dedicated to patients with COVID-19 in Italy.^6^ Studies report high patient satisfaction with remote care offered during the pandemic,^7,8^ and some health care organizations will continue to offer these care modalities after the pandemic, parallel to traditional care.^9^

This alternative model is a collection of care delivery mechanisms that could be used to improve care for some patients with chronic illness, under some circumstances.^10^ To leverage the lessons learned in the pandemic, we must seek patients’ perspectives regarding which alternative care modalities they want to incorporate into their future care and under which circumstances. To address these questions, we conducted a survey to quantify the ideal balance of alternative and traditional care modalities and to describe how patients with chronic illness envision the ideal postpandemic care.

## Methods

### Participants

In this survey study, we recruited a nonprobability sample of adults (≥18 years of age) with any chronic condition (ie, any condition requiring health care for ≥6 months). Patients were recruited from ComPaRe,^11^ a nationwide e-cohort of 47 000 patients with chronic conditions in France who donate time to participate in research. We invited 5999 recently active members of the e-cohort (ie, members who had logged on to their account on the ComPaRe platform in the 6 months before January 2021) to participate in the survey via email, which was conducted from January 27 to February 23, 2021. ComPaRe was approved by the institutional review board of Hôtel-Dieu Hospital in France. Patients provided informed consent. The study followed the American Association for Public Opinion Research (AAPOR) reporting guideline.

### Survey Design

This survey study used an online questionnaire structured in 2 parts. First, we asked patients about their views of the ideal balance among 3 alternative care modalities implemented during the pandemic (teleconsultations, online symptom-checkers, and remote monitoring) and traditional care modalities and about the circumstances in which the use of these alternative care modalities to replace traditional care is considered appropriate. Second, we used open-ended questions to elicit a description of participants’ ideal care, inspired by the alternative care implemented during the COVID-19 pandemic. The survey was conducted in French. We present an English translation of the survey in eMethods 1 in the Supplement.

The questions were framed according to techniques used in psychotherapy to encourage rich answers from participants.^12^ The survey was introduced with a video listing examples of alternative care modalities used in the pandemic. These examples were identified by 1 author (T.O.) by reviewing systematic reviews on changes in care during the pandemic. The review process is described in eMethods 2 in the Supplement. The survey was codeveloped with 3 patients, who participated in semistructured cognitive interviews. It was pilot tested with 4 different patients. Survey development is described in eMethods 3 in the Supplement. The study protocol was preregistered on Open Science Framework.^13^

### Balance Between Alternative Care Modalities and Traditional Care Modalities

In this part of the study, patients were asked to indicate the ideal proportion for which they would use 3 alternative care modalities, replacing the traditional care equivalent: teleconsultations (instead of in-person consultations), online symptom-checkers to identify the right course of action for new symptoms (instead of contacting one’s physician), and remote monitoring to adapt treatment outside consultations (instead of sharing monitoring data during consultations) (eg, “We would like to know what the ideal balance would be for you, between teleconsultations and in-person consultations. For what proportion of your future consultations would you choose to use teleconsultations?”). Responses used 0% to 100% rating scales, with 0% indicating using alternative care modalities for none of one's future care and 100% indicating using alternative care modalities for all of one's future care. An open-ended question asked patients why they selected the specific proportion.

For each of the 3 questions, we calculated the median proportion at which ideal care would consist of the alternative care modality and the proportion of participants whose ideal care consists primarily of the alternative modality (ie, response >50%), primarily of the traditional modality (>5% to ≤50%) and entirely of the traditional modality (≤5%). We assessed the association between this proportion and patient characteristics using linear models (age, educational level, satisfaction with income, multimorbidity, years since diagnosis, previous use of the alternative care modality, Burden of Treatment questionnaire score,^14^ and presence of the most frequently reported conditions, ie, endometriosis, diabetes, high blood pressure, asthma, cancer, and depression). We used univariate models to identify independent variables to enter in generalized linear models, fit in the complete case data set. Statistical significance was set at a 2-sided *P* = .05. Analyses were conducted using R, version 4.0.2 (R Foundation for Statistical Computing).^15^

With the aim of making our findings generalizable to the population of patients with chronic conditions in France, we performed analyses on a weighted data set with calibration on margin. Calibration on margin relies on contingency tables of demographic variables to adjust the margins from sample estimates to the margins of the population. To create the margins matrix, we obtained the proportions of people with chronic illness in France by sex, age categories (<24, 25–34, 35–44, 45–54, 55–64, 65–74, and >75 years), and educational level (lower, middle school or equivalent, high school or equivalent, associate’s degree, undergraduate, or higher education) from the 2017 report of the statistics department of the French public administration Direction de la recherche, de l’évaluation et des statistiques.^16^ The Icarus package in R was used to adjust data sample weights iteratively for the aforementioned variables using raking.^17^

Answers to the open-ended questions were analyzed using inductive content analysis.^18^ We coded participants’ responses with the aim to identify the circumstances in which each alternative care modality was considered an appropriate replacement of traditional care. A preliminary coding scheme was developed by 1 of the authors (T.O.) based on analysis of the first 250 responses and the literature.^19^ This coding scheme was reviewed by another author (D.B.), who used it to independently code 25% of the 250 responses. The authors compared codes and arrived at a consensus for the preliminary coding scheme, which was then used to code all remaining responses (T.O.). New codes were created as needed, 20% of the data set was independently coded as quality control (D.B.), and the authors resolved discrepancies. When all data were coded, codes that described similar concepts were clustered (T.O., D.B. and V.T.T.). For additional information, see the living codebook of the study.^13,20^ We used a predictive modeling method to estimate the degree of data saturation for each question^21^ to determine the number of additional responses that would have to be analyzed to detect 1 additional code.

### Patients’ Description of Ideal Care

Participants answered 2 open-ended questions that aimed to elicit their perspective regarding ideal care as well as specific suggestions as to how to achieve that ideal. We followed the content analysis process outlined above to code responses for 2 prespecified variables: attributes of ideal care and suggested use of alternative care modalities implemented during the COVID-19 pandemic to achieve ideal care.

## Results

Of the 5999 invited individuals, 1529 (mean [SD] 50.3 [14.7] years; 1072 [70.1%] female) agreed to participate (participation rate, 25.5%) (Table 1; eFigure 1 in the Supplement; nonrespondent characteristics are presented in eTable 1 in the Supplement). The most common conditions were endometriosis (303 [19.8%]) and hypertension (266 [17.4%]). A total of 1062 participants (69.5%) experienced multimorbidity (ie, ≥2 chronic conditions).

**Table 1.** Participant Characteristics in the Unweighted and Weighted Sample^a^

Characteristic	Unweighted sample (n = 1529)	Weighted sample (n = 1529)
Sex		
Female	1072 (70.1)	808 (52.8)
Male	457 (29.9)	721 (47.2)
Age, mean (SD), y	50.3 (14.7)	55.2 (17.0)
Educational level		
Lower education	44 (2.9)	149 (9.7)
Middle school or equivalent	148 (9.7)	862 (56.4)
High school or equivalent	226 (14.8)	211 (13.8)
Associate’s degree	323 (21.1)	134 (8.8)
Undergraduate or graduate degree	788 (51.5)	173 (11.3)
Feeling about household income^b^		
Finding it very difficult on present income	36 (2.4)	35 (2.3)
Finding it difficult on present income	145 (9.5)	172 (11.2)
Coping on present income	695 (45.5)	809 (52.9)
Living comfortably on present income	513 (33.6)	368 (24.1)
No. of chronic conditions, median (IQR)	2.0 (1.0-4.0)	2.0 (1.0-4.0)
Multimorbidity	1062 (69.5)	1057 (69.1)
Self-reported diagnosis^c^		
Endometriosis	303 (19.8)	180 (11.8)
High blood pressure	266 (17.4)	307 (20.1)
Depression	149 (9.7)	151 (9.9)
Diabetes	148 (9.7)	166 (10.9)
Asthma	130 (8.5)	105 (6.9)
Cancer	114 (7.5)	146 (9.5)
Time since first diagnosis, median (IQR), y	16.0 (6.0-28.0)	17.0 (7.0-29.0)
Total score on the treatment burden questionnaire, median (IQR)^d^	55.0 (29.0-80.0)	51.0 (25.0-80.0)
Has used teleconsultations^e^	792 (51.8)	741 (48.5)
Has used online symptom-checkers^d^	258 (16.9)	235 (15.4)
Has used remote monitoring^d^^,^^e^	198 (12.9)	215 (14.0)

^a^ Weighted data were obtained after calibration on margins for sex, age, and educational level by using data from a national census describing the French population with chronic conditions.

^b^ Sample sizes were 1389 in the unweighted groups and 1384 in the weighted group.

^c^ Nonexhaustive list. Some participants reported multiple conditions.

^d^ Data were missing in 127 participants.

^e^ Only participants who use monitoring to manage their condition were eligible to answer this question (n = 636 in the unweighted data set and n = 669 in the weighted data set).

### Balance Between Alternative and Traditional Care Modalities

#### Use of Teleconsultations Instead of In-person Consultations

**Figure 1.**
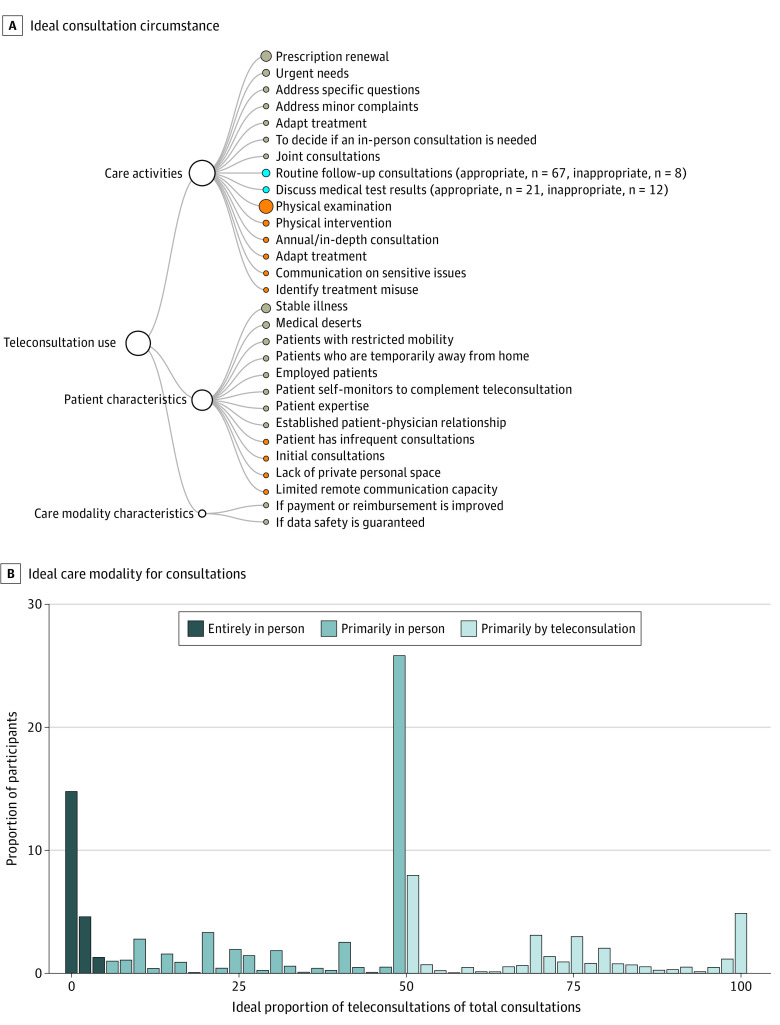
Ideal Proportion and Perceived Appropriate Uses of Teleconsultations A, Circumstances in which participants consider teleconsultations to be an appropriate (gray nodes) or inappropriate (orange nodes) replacement for in-person consultations. The blue nodes indicate circumstances that were reported as both appropriate and inappropriate by different study participants. The number of participants with conflicting opinions is reported in the parentheses. B, Proportion of participants who would, ideally, conduct their future consultations entirely in person (ideal proportion of teleconsultations, 0%-5%), primarily in person (ideal proportion of teleconsultations, 6%-50%), or primarily by teleconsultation (ideal proportion of teleconsultations, >50%).

The circumstances in which teleconsultations were considered appropriate vs inappropriate by patients are presented in Figure 1, Table 2, and eTable 4 in the Supplement. Briefly, teleconsultations were considered appropriate for most routine care activities that do not require physical examination (eg, prescription renewal and discussing checkup results), for patients with mobility or time restrictions attributable to their condition or life circumstances (eg, full-time employment), and who have an established diagnosis, a stable condition, and an established patient-physician relationship.

**Table 2.** The 15 Most Frequent Suggestions for the Appropriate and Inappropriate Uses of Alternative Care Modalities as a Replacement of the Traditional Care Equivalent as Perceived by 1529 Patients With Chronic Illness^a^

Use	Quotations
**Care activities**	
Appropriate for prescription renewal	“For a simple consultation to renew a prescription, teleconsultations are a great tool. But for more complex problems, being face-to-face with our physician is better.” (woman, 39 y, teleconsultation)
Appropriate to rapidly appraise urgency	“It could be practical to know quickly if there is a reason to worry or not.” (woman, 24 y old, online symptom checker)
	“Yes, if it was a chronic condition for which the follow-up is already in place and if the symptoms were not too worrisome, [the online symptom checker] allows us to avoid a useless consultation and to feel reassured when symptoms appear.” (woman, 60 y, online symptom checker)
Appropriate for adapting treatment	“It’s reassuring both for the patient and the physician (for example, [it shows] if the medication is well-tolerated and not rejected [by the patient] and other incidents).” (man, 84 y, remote monitoring)
Appropriate for routine follow-up consultations	“The essence of my contacts with my specialists are the discussion -not the exams (exams such as blood tests and radiology are done separately). Most of the time, physicians just read the exam results while I’m there, then we have a brief discussion, which could absolutely be done by teleconsultation. Being there in person does not add much value.” (man, 58 y, teleconsultation)
Appropriate when other types of care are unavailable (eg, on the weekend or at night)	“I’d first use a symptom checker before calling my doctor, if one for diseases other than covid was available, because experiencing pain often makes us panic and we need to calm down, so any tool that can help us rationalize and re-contextualize the pain is good, because our professional caregivers are not always available and nights can feel long sometimes, so I’d take anything that can help.” (woman, 36 y, online symptom checker)
Appropriate for urgent needs	“Teleconsultations could be used in specific, urgent cases. which I try to avoid experiencing. [I prefer] in-person consultations for all normal occasions, because the personal contact is part of care for me.” (woman, 41 y, teleconsultation)
Inappropriate for urgent needs	“In a situation where I do not feel like I am at major risk, I’d be satisfied with such a tool that can quickly orientate me toward the right care modality. But if I have symptoms that feel critical, I would opt for a real consultation because I know that it’s impossible to replace a global appraisal by a good doctor with a list of non-exhaustive, quick questions from this digital tool. If the tool was perfectly exhaustive though, I’d consult it much more often.” (woman, 36 y, online symptom checker)
Inappropriate for physical examinations^a^	“Every other consultation should be done in person for the patient-physician relationship and to measure [patients’] blood pressure, weight, blood tests, etc.” (woman, 75 y, teleconsultation)
**Patient characteristic**	
Appropriate for patients requiring closer follow-up than that offered by traditional care	“I got to evaluate this tool through the example of a young pregnant woman in my family. It seems to work very well for those who need to follow their data more closely. This is not my case. The occasional medical tests suffice.” (woman, 65 y, remote monitoring)
Appropriate for patients with restricted mobility^b^	“No need to wait seated on hard, uncomfortable chairs. Sitting down can be very painful for me, being home where it’s warm and quiet is much more pleasant. I have managed to keep my appointments even when I was having a crisis, I’d have cancelled these appointments if I had to get to the clinic, because transport + waiting on the chair would have been too difficult and it would have taken me time to recover afterwards.” (woman, 42 y, teleconsultation)
Appropriate for regions with few available health care professionals	“The reference center where I’m followed up for my endometriosis is more than 100 km from my place.” (woman, 36 y, teleconsultation)
Appropriate for stable condition	“When there is nothing new, no change, teleconsultations are largely sufficient and they save us time.” (woman, 54 y, teleconsultation)
Appropriate for conditions the symptoms of which can be observed and reported by patients	“I may not notice some symptoms that would alert a professional to an urgent issue. This has already happened in the past, and it could have been fatal.” (woman, 31 y, online symptom checker)
Inappropriate for patients prone to anxiety regarding their health^c^	“It’s a great tool for well-informed patients, but it could be harmful for those who pay too much attention to themselves or are hypochondriacs.” (man, 57 y, online symptom checker)
**Care modality characteristics**	
Appropriate if the tool is supervised by a physician^d^	“[I would use symptom-checkers] only if my doctor was sent a notification in case of symptoms or behaviors that warrant one.” (man, 35 y, online symptom checker)

^a^ Patients’ appraisal of the need for physical exams is subjective.

^b^ Refers to restrictions attributable to a health condition, including pain and fatigue.

^c^ Patients may overestimate the gravity of their symptoms.

^d^ Supervision refers to the physician reviewing the results of the symptom-checker, either as needed or irrespective of the symptom-checker’s result, and to the need for physicians to commit to view remote monitoring data.

**Figure 2.**
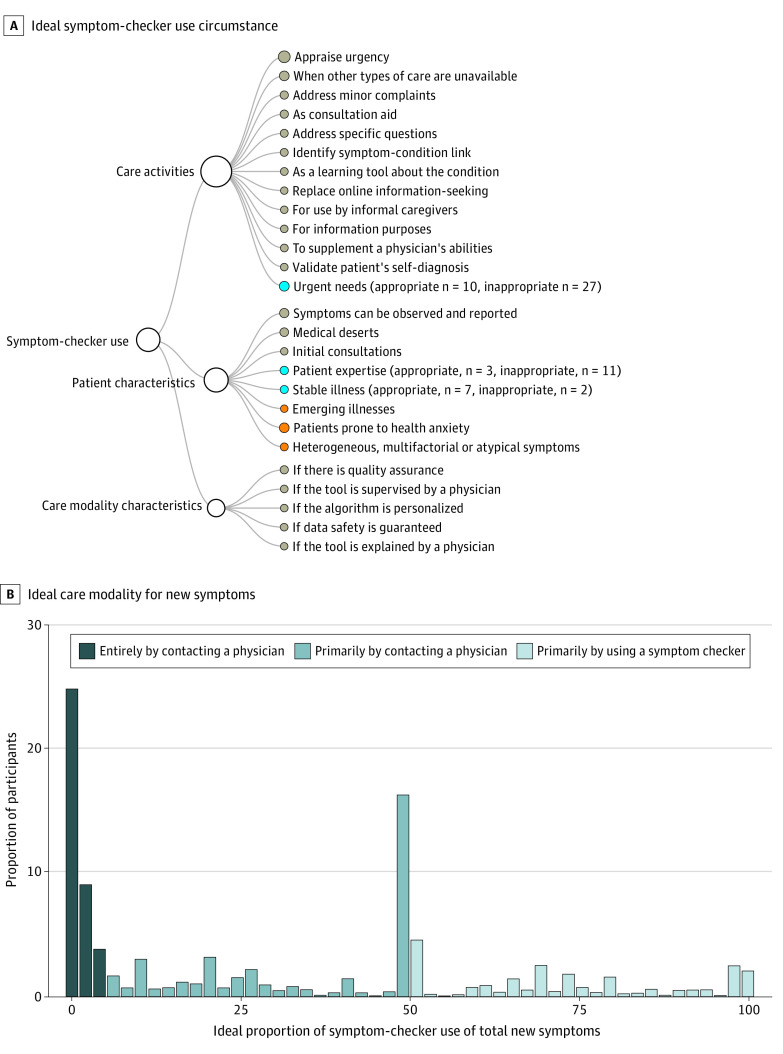
Ideal Proportion and Perceived Appropriate Uses of Online Symptom-Checker Use A, Circumstances in which participants consider using online symptom-checkers to identify the right course of action when new symptoms appear to be an appropriate (gray nodes) or inappropriate (orange nodes) replacement for contacting their physician. The blue nodes indicate circumstances that were reported as both appropriate and inappropriate by different study participants. For these nodes, the number of participants with conflicting opinions is reported in the parentheses. B, Proportion of participants who would, ideally, react to the appearance of new symptoms in the future entirely by contacting a physician (ideal proportion of symptom-checker use, 0%-5%), primarily by contacting a physician (ideal proportion of symptom-checker use, 6%-50%), or primarily by using symptom-checkers (ideal proportion of symptom-checker use, >50%).

Participants would use teleconsultations instead of in-person consultations for 50.0% of all their future consultations (IQR, 11.0%-52.0%) (Figure 2; eTable 2 in the Supplement). Ideally, consultations would be entirely in person for 312 participants (20.4%), primarily in person for 719 (47.0%), and primarily remote for 477 (31.2%). In univariate models, prior teleconsultation use was the only independent variable, with a significant association with the outcome (β = 18.0; 95% CI, 11.8-24.2; *P* < .001) (eTable 3 in the Supplement).

#### Use of Online Symptom-Checkers Instead of Physician Contact When New Symptoms Appear

Participants would use online symptom-checkers instead of contacting their physician for 22.0% of the times that new symptoms appear (IQR, 2.0%-50.0%). Ideally, 564 participants (36.9%) would appraise new symptoms entirely by contacting their physician, 574 (37.5%) primarily by contacting their physician, and 357 (23.4%) primarily by using online symptom-checkers. Two variables with significant association to the outcome were identified: asthma (β = −12.4; 95% CI, −21.9 to −2.8; *P* = .01) and endometriosis (β = −8.5; 95% CI, −14.6 to −2.3; *P* = .007) (eTable 3 in the Supplement).

Online symptom-checkers were considered appropriate as decision aids for patients to decide whether an emergency consultation is warranted, for addressing minor, nonurgent ailments, for use at times and places with poor physician availability (eg, on weekends), as a preconsultation tool for patients to collect information for the subsequent consultation, and for newly diagnosed patients without expertise in managing their condition (Figure 2 and Table 2; eTable 4 in the Supplement). Symptom-checkers are inappropriate for patients prone to health anxiety and patients with heterogeneous symptoms, atypical of their condition, or symptoms that cannot be reported without help from a physician. Patients’ main requirements for appropriate symptom-checker use were quality assurance (eg, accreditation by a relevant governing body) and supervision of symptom-checker results by a physician.

#### Use of Remote Monitoring Instead of Sharing Monitoring Data in Consultations to Adapt Treatment

Participants would use remote monitoring to adapt their treatment outside consultations instead of in consultations 52.3% of the time (IQR, 25.5%-85.4%). Ideally, sharing monitoring data to adapt one’s treatment is done entirely in consultations for 100 of 669 participants (14.9%) who used health monitoring and were eligible to answer the question, primarily in consultations for 192 (28.7%), and primarily remotely for 377 (56.4%). A significant, negative association was found with having cancer (β = −27.1; 95% CI, −43.4 to −10.8; *P* = .001) and significant, positive associations with level of education (middle school: β = 32.0; 95% CI, 12.8-51.3; *P* = .001; high school: β = 32.0; 95% CI, 13.8-50.3; *P* < .001; associate’s degree: β = 31.5; 95% CI, 12.9-50.0; *P* < .001; and or undergraduate degree and above: β = 31.0; 95% CI, 12.7-49.3; *P* < .001 [reference category, lower educational level), satisfaction with income (comfortable as compared with very difficult situation: β = 40.9; 95% CI, 9.9-71.9; *P* = .009), and endometriosis (β = 16.2; 95% CI, 7.2-25.2; *P* < .001) (eTable 3 in the Supplement).

**Figure 3.**
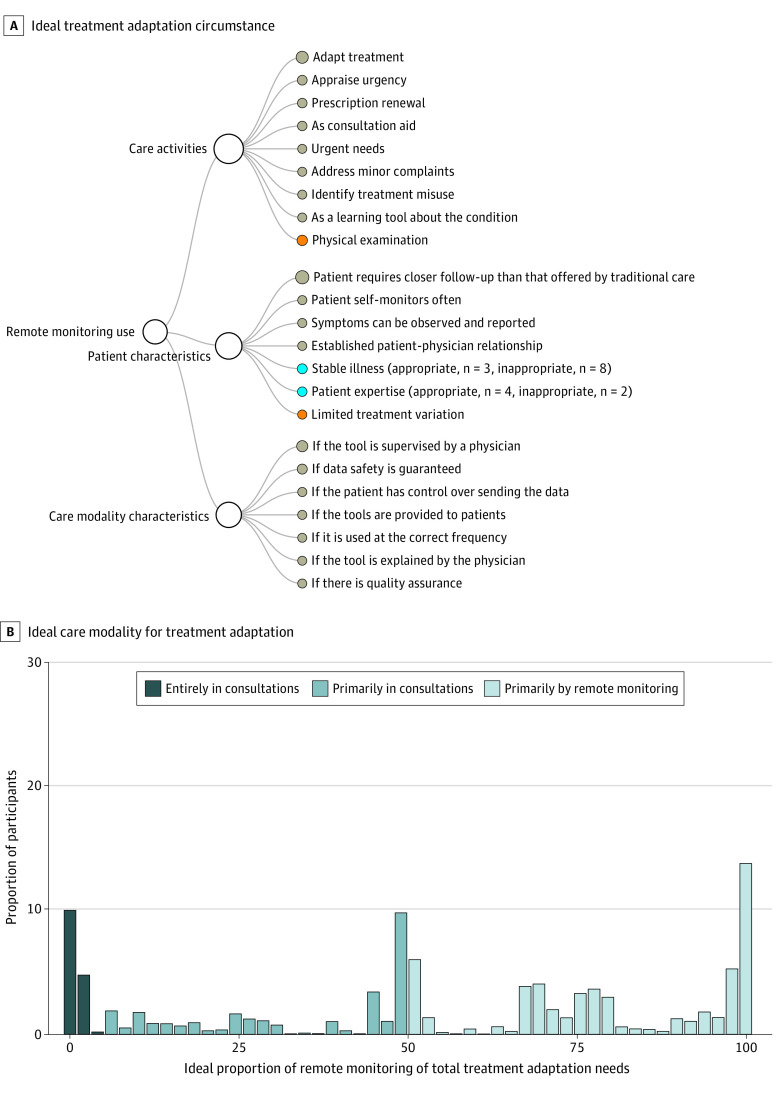
Ideal Proportion and Perceived Appropriate Uses of Remote Monitoring A, Circumstances in which participants consider remote monitoring for treatment adaptation outside consultations to be an appropriate (gray nodes) or inappropriate (orange nodes) replacement for adapting their treatment after revising monitoring data in consultations. The blue nodes indicate circumstances that were reported as both appropriate and inappropriate by different study participants. For these nodes, the number of participants with conflicting opinions is reported in the parentheses. B, Proportion of participants who would, ideally, have their treatment adapted entirely in consultations (ideal proportion of remote monitoring, 0%-5%), primarily in consultations (ideal proportion of remote monitoring, 6%-50%), or primarily outside consultations by using remote monitoring (ideal proportion of remote monitoring, >50%).

Remote monitoring is appropriate for renewing prescriptions, adapting treatment rapidly, and assessing whether medical help is needed for patients with unstable conditions who need or prefer closer follow-up than that offered by traditional care and whose symptoms do not require physical examination (Figure 3 and Table 2; eTable 4 in the Supplement). Patients’ main requirement was that monitoring data would be supervised by their physician.

### Patients’ Description of Postpandemic Care

We identified 22 attributes of ideal care (eTable 5 in the Supplement), including *lean* (ie, without components that provide no value to patients) (432 [28.2%]) and *responsive t*o patients’ needs as opposed to following a one-size-fits-all schedule (206 [13.5%]). Ideal care would be, at least partially, in person for 199 participants (13.0%), and 143 (9.4%) imagined ideal care would be the same as prepandemic care (eg, because they were satisfied with their prepandemic care or did not consider that improvement was feasible). Participants reported 114 uses of alternative care modalities to achieve ideal care (eTable 5 and eFigure 2 in the Supplement).

#### Use of Teleconsultations in the Right Circumstances

Participants suggested broader use of teleconsultations (594 [38.9%]), particularly for circumstances in which the patient’s physical presence at the clinic does not add value to their care (eg, 283 [18.5%] suggested doing prescription renewals via teleconsultations). Other than reducing travel, participants explained that teleconsultations provided value because they can be scheduled more quickly than in-person consultations and allow for more regular contact with the physician.

#### Replacement of Consultations With Other Communication Modalities

Participants suggested that consultations are not the right care modality for many care activities. They proposed renewing prescriptions without consultation (eg, based on laboratory test results communicated by email in 43 [2.8%]) and using dynamic care modalities to address issues that arise between consultations (eg, remote monitoring in 66 [4.3%] and brief patient-physician communication via email or mini-teleconsultations in 135 [8.8%]).

#### Breaking of the Rules for Less Disruptive Care

Some of the suggestions concerned breaking care rules. For example, prolonging prescription validity (10 [0.6%]) can reduce consultations and pharmacy visits. Booking consultations via scheduling websites (54 [3.5%]) as opposed to calling the physician offers convenient functions (eg, alerts when earlier consultations open up). Some participants suggested that consultations should not be scheduled at prespecified intervals but be contingent on the result of remote screening (5 [0.3%]).

#### Connection of All Caregivers in the Patients’ Network

Remote technologies can facilitate communication within the patient’s care network, sparing the patient the burden of information sharing between caregivers (eg, asynchronous communication between the patient’s physicians to check medication compatibility in 25 [1.6%] and synchronous, joint teleconsultations with multiple physicians in 5 [0.3%]).

#### Centralization of Patient Care

A single health record per patient (34 [2.2%]) would be shareable with caregivers, who would consult and update the record to avoid information loss. For patients with multimorbidities, care could be managed by a single caregiver (13 [0.8%]), who could remotely consult specialists on behalf of the patient (25 [1.6%]).

## Discussion

In this survey study, 1529 patients rated their ideal balance of alternative and traditional care modalities, proposed 67 criteria for the appropriateness of the future use of alternative care modalities, and suggested 114 uses of alternative care modalities to achieve their ideal care. Patients would use alternative care modalities at least some of the time, depending on their health status, constraints, and preferences and on the type of care activity they seek to obtain. Previous studies^7,8,22^ report that patients want to continue using remote care modalities after the pandemic. A survey of preferences for preoperation consultations showed that the perceived appropriateness of teleconsultations varies, depending on care activities.^22^

### Implications for Research and Care

Our findings show that we already have at our disposal many of the tools needed to improve care. Deciding which tools should be used for which patient depends on care activity and patient characteristics. First, patient-physician dyads can use these characteristics to decide how remote care modalities could fit in the patient’s care. Second, some participants in our study envisioned using remote care modalities not to replace traditional care but to supplement it (eg, using teleconsultations for more frequent follow-up). This use differs from the intended use of these tools as envisioned by developers and clinicians. Third, studies^23,24^ have used fixed patient characteristics to predict the uptake of remote care. Future studies should assess the association of the time-varying criteria identified in our study with uptake. Fourth, it has been proposed that after the pandemic, care avoidance should be mitigated.^25^ The appropriate level of contact with the health care system should be codefined with patients to ensure that what patients view as sensible care use is not misinterpreted as avoidance. Fifth, patients’ preference for alternative care modalities may be affected by cost. The French universal health insurance system reimburses teleconsultations at the same rate as in-person consultations. Novel care modalities would require determining the pricing and reimbursement of these services. Changes in the reimbursement of remote care have already been implemented in the wake of the pandemic.^26^ Sixth, some of the ideas proposed by participants may be easier to implement in clinical practice than others. For example, prolonging prescription validity could be implemented relatively easily for some medication classes but not for others (eg, opioids). Other ideas might require substantial changes in infrastructure (eg, developing a shared medical record platform).

### Strengths and Limitations

This study has several strengths. This is the first study, to our knowledge. to examine patients’ vision of postpandemic care and identify appropriate uses of alternative care modalities. We analyzed responses from 1529 patients. The model used to predict data saturation indicates that if we doubled the number of participants in our study, we would have identified no additional appropriate and inappropriate uses for teleconsultations, 2 additional uses for symptom-checkers, and 3 for remote monitoring (eFigure 3 in the Supplement). Our diverse sample included older and multimorbid patients and was weighted to reflect the general population of chronically ill patients more closely.

This study also has limitations. Because ComPaRe is an e-cohort, all participants have internet access. This survey may overestimate patients’ access to technology-based care. However, 83% of the French population uses the internet.^27^ Responses may be affected by characteristics of the French health care system (eg, universal health insurance). We did not examine the association between ethnicity and willingness to use remote care because of regulatory restrictions regarding the collection of ethnicity data in France.^28^ A systematic review^29^ found mixed findings regarding the association between ethnicity and teleconsultation use in general practice during the pandemic. Our results may not be generalizable to nonfrancophone immigrants. Despite weighting, our sample is not perfectly representative of the general population of patients with chronic illness regarding the prevalence of specific conditions (eg, endometriosis), which may limit the generalizability of our findings. Study respondents were more likely to experience multimorbidity and hypertension and less likely to have endometriosis than nonresponders. Finally, some suggestions identified in the qualitative analysis were reported by a small number of participants.

## Conclusions

This study presents the views of 1529 patients regarding the appropriate use of alternative care modalities inspired from the pandemic and the ideal balance between alternative and traditional care modalities. These findings provide a guide for redesigning care in collaboration with patients after the pandemic.

## Notes

Supplement. **eMethods 1.** Survey (English Translation) **eMethods 2.** Literature Review **eMethods 3.** Description of Survey Development and Piloting **eFigure 1.** Flow Chart **eFigure 2.** Suggested Uses of Alternative Care Modalities Implemented During the Pandemic in Post-Pandemic Care **eFigure 3.** Cumulative Code Accumulation Curve Representing Data Saturation **eTable 1.** Characteristics of Responders and Non-responders (Unweighted Sample) **eTable 2.** Quantitative Outcomes Indicating the Ideal Use of Alternative Care Modalities as a Proportion of Total Relevant Care Needs, in the Unweighted and Weighted Sample **eTable 3.** Results of Linear Models in the Weighted Sample **eTable 4.** Code List for the Appropriate and Inappropriate Uses of Alternative Care Modalities **eTable 5.** Code List for the Attributes of Ideal Care and Use of Alternative Care Modalities to Achieve Ideal Care
