# Supplementary material for: Preferences for Alternative Care Modalities Among French Adults With Chronic Illness
Source: JAMA Netw Open. 2021 Dec 29;4(12):e2141233. doi: 10.1001/jamanetworkopen.2021.41233 (PMC8717105; doi:10.1001/jamanetworkopen.2021.41233)
Supplement: Supplement. — eMethods 1. Survey (English Translation) eMethods 2. Literature Review eMethods 3. Description of Survey Development and Piloting eFigure 1. Flow Chart eFigure 2. Suggested Uses of Alternative Care Modalities Implemented During the Pandemic in Post-Pandemic Care eFigure 3. Cumulative Code Accumulation Curve Representing Data Saturation eTable 1. Characteristics of Responders and Non-responders (Unweighted Sample) eTable 2. Quantitative Outcomes Indicating the Ideal Use of Alternative Care Modalities as a Proportion of Total Relevant Care Needs, in the Unweighted and Weighted Sample eTable 3. Results of Linear Models in the Weighted Sample eTable 4. Code List for the Appropriate and Inappropriate Uses of Alternative Care Modalities eTable 5. Code List for the Attributes of Ideal Care and Use of Alternative Care Modalities to Achieve Ideal Care [file jamanetwopen-e2141233-s001.pdf]

Supplementary Online Content

Oikonomidi T, Ravaud P, Barger D, Tran V-T. Preferences for alternative care modalities among French adults with chronic illness. *JAMA Netw Open*. 2021;4(12):e2141233. doi:10.1001/jamanetworkopen.2021.41233

eMethods 1. Survey (English Translation)

eMethods 2. Literature Review

eMethods 3. Description of Survey Development and Piloting

eFigure 1. Flow Chart

eFigure 2. Suggested Uses of Alternative Care Modalities Implemented During the Pandemic in Post-Pandemic Care

eFigure 3. Cumulative Code Accumulation Curve Representing Data Saturation

eTable 1. Characteristics of Responders and Non-responders (Unweighted Sample)

eTable 2. Quantitative Outcomes Indicating the Ideal Use of Alternative Care Modalities as a Proportion of Total Relevant Care Needs, in the Unweighted and Weighted Sample

eTable 3. Results of Linear Models in the Weighted Sample

eTable 4. Code List for the Appropriate and Inappropriate Uses of Alternative Care Modalities

eTable 5. Code List for the Attributes of Ideal Care and Use of Alternative Care Modalities to Achieve Ideal Care

This supplementary material has been provided by the authors to give readers additional information about their work.

© 2021 Oikonomidi T et al. *JAMA Network Open.*

eMethods 1: Survey (English translation)

--- Home page ---

During the COVID-19 pandemic, the ways in which patients access health care services changed and new, technology-based services emerged, aiming to limit the spread of the virus. **These changes may affect the way we access health care even after the end of the COVID-19 pandemic.**

For example, a hospital that replaced in-person consultations with teleconsultations in 2020 may decide to offer **both types of consultations to their patients** after the end of the pandemic.

This survey aims to understand how we can adapt regular care (that is, the way you used to receive care before the pandemic started), by adopting components from the care models applied during the pandemic. First, we will present you a list of things that changed in health care delivery during the pandemic in a short video. You will then be asked questions about how we could best combine these new care components with regular care in order to create the ideal care model for you.

Participation in this survey takes 15 to 20 minutes.

---Page 1 ---

Your vision of the future of health care

In the video below, we present examples of changes in health care delivery that took place during the pandemic. To see a written list of these changes, click here: https://inspire-compare.fr/compare/Window_after_interviews.pdf

[embedded video https://youtu.be/GbfOYypjmn0]

Imagine that all these innovations put in place during the pandemic remain available to you, in the long-term, after the end of the pandemic. How could we integrate these innovations (if at all) in the care of patients with chronic illnesses in order to improve health care after the end of the pandemic?

The innovations presented here are only a few of the modifications in health care delivery experienced by patients during the pandemic. Do not hesitate to express your own ideas in your answers to the questions below. Do not hesitate to give detailed responses so that we can better understand your point of view.

Imagine the ideal care for yourself, in the long term. By ideal care we mean the care you wish to receive as a patient, according to your own criteria (e.g., more effective, less burdensome, etc.).

1. In which ways would it be **different** from the regular care you received before the pandemic? *
2. How would the innovations in health care delivery, implemented during the pandemic and presented above help you obtain this ideal care? *

--- Page 2 ---

Consultations with your physician

In the following pages of this survey, we describe 3 changes in health care delivery experienced by many patients during the pandemic.

The following questions may help you generate more ideas about the future of health care. In this case, do not hesitate to **return to the previous page of the survey and modify your responses.**

During the pandemic, many patients replaced **in-person consultations** with their physician with **teleconsultations** (e.g., by phone or video call).

a. Have you used teleconsultations in the management of your chronic illness, at least once, **before the pandemic**?*

   ☐ Yes

   ☐ No

b. Did you use teleconsultations in the management of your chronic illness, at least once, **during the pandemic**?*

   ☐ Yes

   ☐ No

Imagine that after the end of the pandemic, you could use teleconsultations for the management of your chronic illness. We would like to know what **the ideal balance** would be for you, between teleconsultations and in-person consultations.

c. For what proportion of your future consultations, would you choose to use **teleconsultations**?*

Your remaining consultations would be in-person.

[0 to 100% sliding scale, labelled: “None of my consultations” to “All of my consultations”]

d. Please use the text box below to explain why you chose this response. [text box]

---Page 3 ---

Managing new or worsening symptoms

During the pandemic, many patients used interactive websites called symptom checkers, to quickly receive advice for new or worsening symptoms they experienced, instead of contacting their physician.

A symptom checker is a website that asks questions about your symptoms and, using an algorithm, gives you advice on how to best manage them (e.g., self-management advice, recommendation to go to the E.R. or call your regular physician, etc.)

a. Had you used a symptom checker at least once **before the pandemic**?*

   ☐ Yes

   ☐ No

b. Did you use a symptom checker at least once, **during the pandemic** (including to identify if the symptoms you experienced could be due to a COVID-19 infection)?*

   ☐ Yes

   ☐ No

© 2021 Oikonomidi T et al. *JAMA Network Open.*

Imagine that after the end of the pandemic, you could use symptom checkers to receive advice at instances of new or worsening symptoms of your illness, instead of having to contact your physician.

c. For what proportion of these instances would you choose to use **a symptom checker**?*

In the remaining instances you would contact your physician.

[0 to 100% sliding scale, labelled: "None of these instances" to "All of these instances"]

d. Please use the text box below to explain why you chose this response. [text box]

---Page 4 ---

Monitoring your health at home

The questions below concern only patients who use self-monitoring tools to monitor and manage their condition (e.g., a glucose meter, a blood pressure cuff, a symptom diary, etc). Do you use such monitoring tools? *

- ☐ Yes
- ☐ No

[Note: the following 2 questions were presented only to participants who selected "Yes" in the above question.]

During the pandemic, some patients shared the data collected using self-monitoring tools with their physician, remotely and outside of regular consultations (e.g., by giving their physician direct access to their dashboard, by sending the data via e-mail). This enabled their physician to adjust their treatment outside of consultations.

a. Had you shared monitoring data remotely with your physician at least once **before the pandemic**? *

   - ☐ Yes
   - ☐ No

b. Did you share monitoring data remotely with your physician at least once **during the pandemic?** *

   - ☐ Yes
   - ☐ No

Imagine that after the end of the pandemic, you could share data collected by using a self-monitoring tool with your physician, remotely, when you need a medical opinion on your data. This would enable your physician to adjust your treatment outside of regular consultations.

c. For what proportion of these instances would you choose to share your data with your physician remotely, outside of consultations? *

In the remaining instances you would share your data with your physician in consultation.

[0 to 100% sliding scale, labelled: "None of these instances" to "All of these instances"]

d. Please use the text box below to explain why you chose this response. [text box]

---Page 5 ---

Finally, of the options below, please select **all that apply to** you:*

- ☐ I am a health care professional (e.g., physician, nurse, physiotherapist, etc.)
- ☐ I am a caregiver to an ill family member or friend
- ☐ I am neither a health care professional nor a caregiver

* Starred questions require a response to continue to the next page of the survey.

eMethods 2: Literature review

We followed standard literature review methods to identify alternative care modalities implemented during the COVID-19 pandemic.

First, we searched Pubmed on December 2, 2020 using the following search strategy:

#7	#4 AND #5 AND #6
#6	#1 OR #2 OR #3
#5	"systematic review"[Publication Type] OR "systematic review"[Text Word]
#4	"covid 19"[Title/Abstract] OR "covid19*"[Title/Abstract] OR "covid 19"[Title/Abstract] OR "SARS CoV-2"[Title/Abstract] OR "2019nCoV"[Title/Abstract] OR "2019 ncov"[Title/Abstract] OR "nCoV2019"[Title/Abstract] OR "nCoV-2019"[Title/Abstract] OR "coronavir*"[Title/Abstract] OR "corona-virus"[Title/Abstract] OR "coronovir*"[Title/Abstract] OR "corono-virus"[Title/Abstract] OR "corona-virus"[Title/Abstract] OR "corono-virus"[Title/Abstract] OR "betacoronavir*"[Title/Abstract] OR "beta-coronavirus"[Title/Abstract] OR "beta-coronavirus"[Title/Abstract] OR "2019 ncov"[Title/Abstract] OR "n-cov"[Title/Abstract] OR "ncov*"[Title/Abstract] OR (("virus"[Title/Abstract] OR "viruses"[Title/Abstract] OR "viral"[Title/Abstract]) AND "wuhan*"[Title/Abstract]) OR (("virus"[Title/Abstract] OR "viruses"[Title/Abstract] OR "viral"[Title/Abstract]) AND "covid*"[Title/Abstract]) OR "COVID-19 diagnostic testing"[Supplementary Concept] OR "covid 19"[Supplementary Concept] OR "severe acute respiratory syndrome coronavirus 2"[Supplementary Concept]
#3	("reorganization"[Title/Abstract] OR "remote*"[Title/Abstract] OR "distance"[Title/Abstract] OR "video*"[Title/Abstract]) AND ("care"[Title/Abstract] OR "healthcare"[Title/Abstract] OR "health care"[Title/Abstract] OR "consult*"[Title/Abstract] OR "appointment*"[Title/Abstract])
#2	("monitoring"[Title/Abstract] OR "surveillance"[Title/Abstract]) AND "remote*"[Title/Abstract])
#1	("social media"[Title/Abstract] OR "Facebook"[Title/Abstract] OR "Whatsapp"[Title/Abstract] OR "Youtube"[Title/Abstract] OR "Skype"[Title/Abstract] OR "Twitter"[Title/Abstract] OR "WeChat"[Title/Abstract] OR "Weibo"[Title/Abstract] OR "SMS"[Title/Abstract] OR "short messaging service"[Title/Abstract] OR "text messages"[Title/Abstract] OR "text messaging"[Title/Abstract] OR "virtual"[Title/Abstract] OR "online"[Title/Abstract] OR "web based"[Title/Abstract] OR "web delivered"[Title/Abstract] OR "web platform"[Title/Abstract] OR "smartphone"[Title/Abstract] OR "internet"[Title/Abstract] OR "wearable*"[Title/Abstract] OR "telephone*"[Title/Abstract] OR "phone*"[Title/Abstract] OR "digital"[Title/Abstract] OR "m health"[Title/Abstract] OR "mhealth"[Title/Abstract] OR "e health"[Title/Abstract] OR "ehealth"[Title/Abstract]

© 2021 Oikonomidi T et al. *JAMA Network Open.*

	OR "conversational agent*"[Title/Abstract] OR "chatbot"[Title/Abstract] OR "artificial intelligence"[Title/Abstract] OR "telecare*"[Title/Abstract] OR "telehealth*"[Title/Abstract] OR "telemedicine*"[Title/Abstract] OR "telepsychiatry"[Title/Abstract] OR "telerehabilitation*"[Title/Abstract] OR "tele-care"[Title/Abstract] OR "tele-health"[Title/Abstract] OR "tele-medicine"[Title/Abstract] OR "tele-psychiatry"[Title/Abstract] OR "tele-rehabilitation"[Title/Abstract]<br><br>OR "technolog*"[Title/Abstract] OR "self triage"[Title/Abstract] OR "symptom checker"[Title/Abstract] OR ("messag*"[Title/Abstract] AND ("platform"[Title/Abstract] OR "software"[Title/Abstract]))

Then, one author (T.O.) selected eligible studies according to the following criteria:

- Inclusion criteria: Systematic reviews, including at least 1 primary study on COVID-19, including at least 1 primary study describing technology-based or non-technology-based reorganization of care.
- Exclusion criteria: Reviews of apps available on smartphone app stores (i.e., not including any studies), reviews including only primary studies on the diagnostic accuracy of technology-based interventions implemented during the COVID-19 pandemic.

After screening, we included the following 8 systematic reviews:

1. Boyce L, Nicolaides M, Hanrahan JG, Sideris M, Pafitanis G. The early response of plastic and reconstructive surgery services to the COVID-19 pandemic: A systematic review. Journal of Plastic, Reconstructive & Aesthetic Surgery. 2020.
2. Davalbhakta S, Advani S, Kumar S, Agarwal V, Bhoyar S, Fedirko E, Misra DP, Goel A, Gupta L, Agarwal V. A systematic review of smartphone applications available for corona virus disease 2019 (COVID19) and the assessment of their quality using the mobile application rating scale (MARS). Journal of medical systems. 2020 Sep;44(9):1-5.
3. Golinelli D, Boetto E, Carullo G, Nuzzolese AG, Landini MP, Fantini MP. Adoption of Digital Technologies in Health Care During the COVID-19 Pandemic: Systematic Review of Early Scientific Literature. Journal of medical Internet research. 2020;22(11):e22280.
4. Hojaij FC, Chinelatto LA, Boog GH, Kasmirski JA, Lopes JV, Sacramento FM. Surgical Practice in the Current COVID-19 Pandemic: A Rapid Systematic Review. Clinics. 2020;75.
5. Monaghesh E, Hajizadeh A. The role of telehealth during COVID-19 outbreak: A systematic review based on current evidence. BMC Public Health. 2020:20.
6. Prakash L, Dhar SA, Mushtaq M. COVID-19 in the operating room: a review of evolving safety protocols. Patient Safety in Surgery. 2020 Dec;14(1):1-8.
7. Tebeje TH, Klein J. Applications of e-Health to Support Person-Centered Health Care at the Time of COVID-19 Pandemic. Telemedicine and e-Health. 2020.

© 2021 Oikonomidi T et al. *JAMA Network Open.*

8. Yue JL, Yan W, Sun YK, Yuan K, Su SZ, Han Y, Ravindran AV, Kosten T, Everall I, Davey CG, Bullmore E. Mental health services for infectious disease outbreaks including COVID-19: a rapid systematic review. Psychological Medicine. 2020 Nov 5:1-6.

The PRISMA flow chart is presented below:

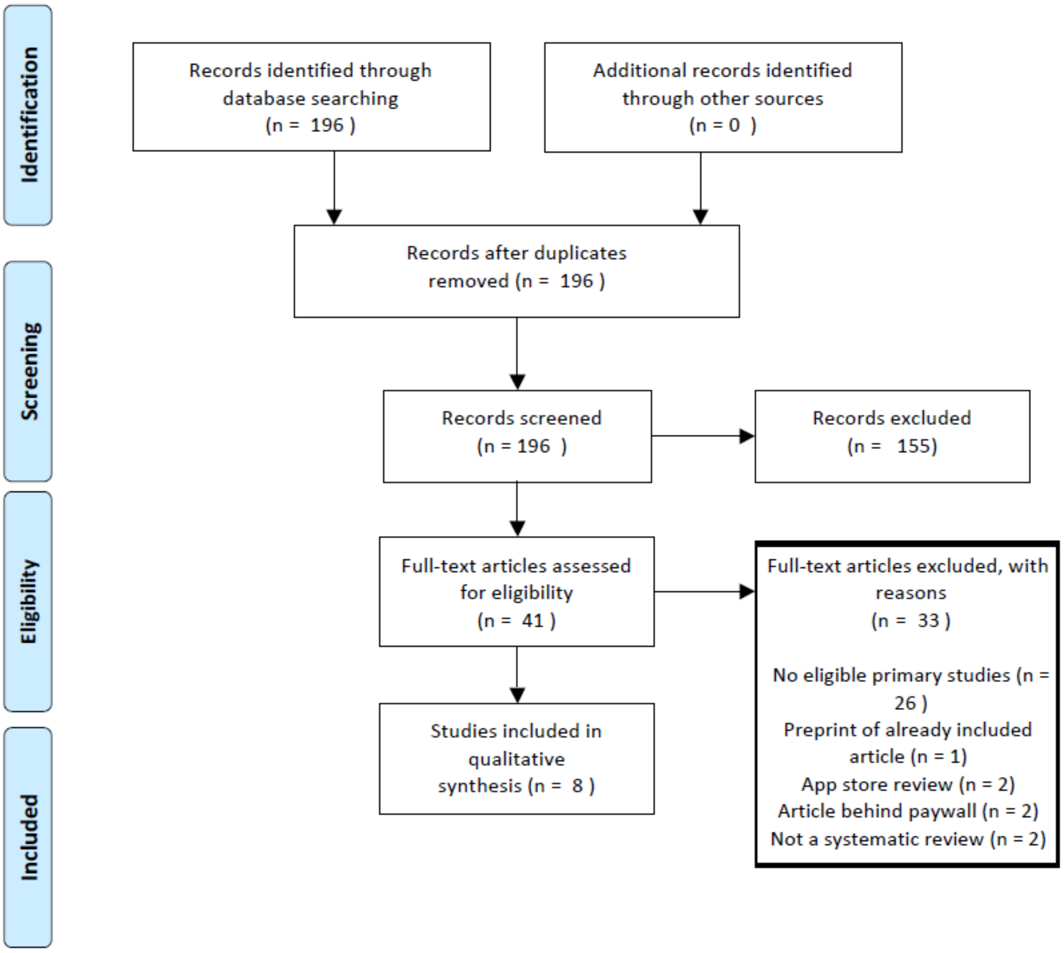

Qualitative data extraction was performed by one author (T.O.) by using content analysis. The author sought to identify components of technology or nontechnology based reorganization of care from primary studies included in the systematic review, by examining the results section and the summary tables of the 8 included reviews. The identified components of reorganization of care were synthesized in a single list by comparing the extracted data across systematic reviews and merging similar components into a single entry. A revised version of this list, written in non-technical language, was presented to the survey participants to illustrate the concept of blended care and to encourage idea generation.

eMethods 3: Description of survey development and piloting

Questions 1-2

Question development

The first two questions were developed based on brief solution-focused therapy counseling techniques. One technique used in solution-focused therapy is visualization of the future at a time when the patient's therapy goal will have been achieved. The counselor can ask questions to bring this ideal future into focus. For example, a patient who comes into therapy with the request to grow their self-confidence may be asked to describe a day in the future, when they will have become self-confident, in great detail (e.g., "How would your self-confidence show in work meetings?", "How will family dinners be different then compared to now?"

Based on this, the authors drafted the following questions:

"Imagine the ideal care for yourself, in the long term. In which ways would it be different from the regular care you received before the pandemic?

Which components of your pre-pandemic care would be carried over to your ideal care?

Which components of your pre-pandemic care would be removed completely or replaced by pandemic-care innovations, to achieve your ideal care?

How would the innovations in health care delivery, implemented during the pandemic and presented above help you obtain this ideal care?"

Question testing in cognitive interviews and piloting

We then conducted cognitive interviews with 3 patients (a 26-year-old woman with major depressive disorder, a 20-year-old woman with type 1 diabetes and generalized anxiety disorder, and a 57-year-old woman with hypothyroidism).

The patients were presented with a draft of the survey on the ComPaRe platform and were asked to complete it while thinking out loud, in the presence of one of the authors (T.O.). At the end of each webpage, the patients were asked standard cognitive interviewing questions (e.g., "What do you think this question is trying to identify?", "Could you rephrase this question, in your own words?"), and were asked to provide any suggestions that could improve the study.

Based on the feedback, we retained the first and last question reported above. Questions two and three produced no original data (i.e., they elicited responses that were repetitive of previous responses). We also decided to merge the two questions:

"Imagine the ideal care for yourself, in the long term. In which ways would it be different from the regular care you received before the pandemic? How would the innovations in health care delivery, implemented during the pandemic and presented above help you obtain this ideal care? "

Finally, the survey was pilot-tested with four participants of the ComPaRe cohort. Pilot-testing replicated the dissemination process of the final survey: the four participants received an email inviting them to participate in the survey, containing a link to their account on the ComPaRe platform, where the survey could be completed. They were additionally asked to time survey completion. After completing the main survey, participants were asked 3 additional questions in a separate webpage: the duration of survey completion, to describe any problems or difficulties they encountered in the survey and to propose additional modifications that, in their view, could improve the survey (open-ended questions).

© 2021 Oikonomidi T et al. *JAMA Network Open.*

Survey completion lasted an average of 21 minutes (range: 5-30 minutes). Participants main difficulty was caused by the term « ideal care », which was not specified further. Participants were unsure whether the term referred to ideal care for themselves, physicians, the care system, patients in general, etc. The question was reworded to specify this. Participants also proposed that the question be broken into two smaller questions. This led to the final version of the question:

"Imagine the ideal care for yourself, in the long term. By ideal care we mean the care you wish to receive as a patient, according to your own criteria (e.g., more effective, less burdensome, etc.).

1. In which ways would it be different from the regular care you received before the pandemic?
2. How would the innovations in health care delivery, implemented during the pandemic and presented above help you obtain this ideal care?"

Questions 3-5

Question development

The following three compulsory questions refer to the use of alternative traditional care modalities. The authors aimed to develop questions that were not vague or general (e.g., "Would you use teleconsultations in the future?"). Inspired by the use of percentages and proportions, which is a common technique used to develop concrete goals in psychotherapy (e.g., "I will commute to work by bike instead of by car 3 days out of 5"), the authors developed the following question format:

"For what proportion of your future consultations, would you choose to use teleconsultations?"

Question testing in cognitive interviews and piloting

Patients provided positive feedback for this question in the cognitive interviews. They found it easy to conceptualize their care as a whole that can be completed partially by using new care modalities. Both the participants and interviewer found that the thinking process that led participants to select a proportion was informative, and should be captured for further analysis. Therefore, the participants and the interviewer agreed to add an optional open-ended question to each of the close-ended questions:

"Please use the text box below to explain why you chose this response."

Pilot-testing did not lead to any changes in questions 3-5.

Cover letter

We drafted a brief cover letter that was sent to participants via e-mail, describing the purpose of the survey, the completion time (determined from piloting the survey with four participants, see below), and a link to the participant's account on the ComPaRe website where they could complete the survey. The cover letter was accompanied by the name and photograph of one of the authors (V.T.T.). The cover letter was presented to participants in cognitive interviewing and piloting. No modifications were proposed by participants.

eFigure 1 : Flow chart

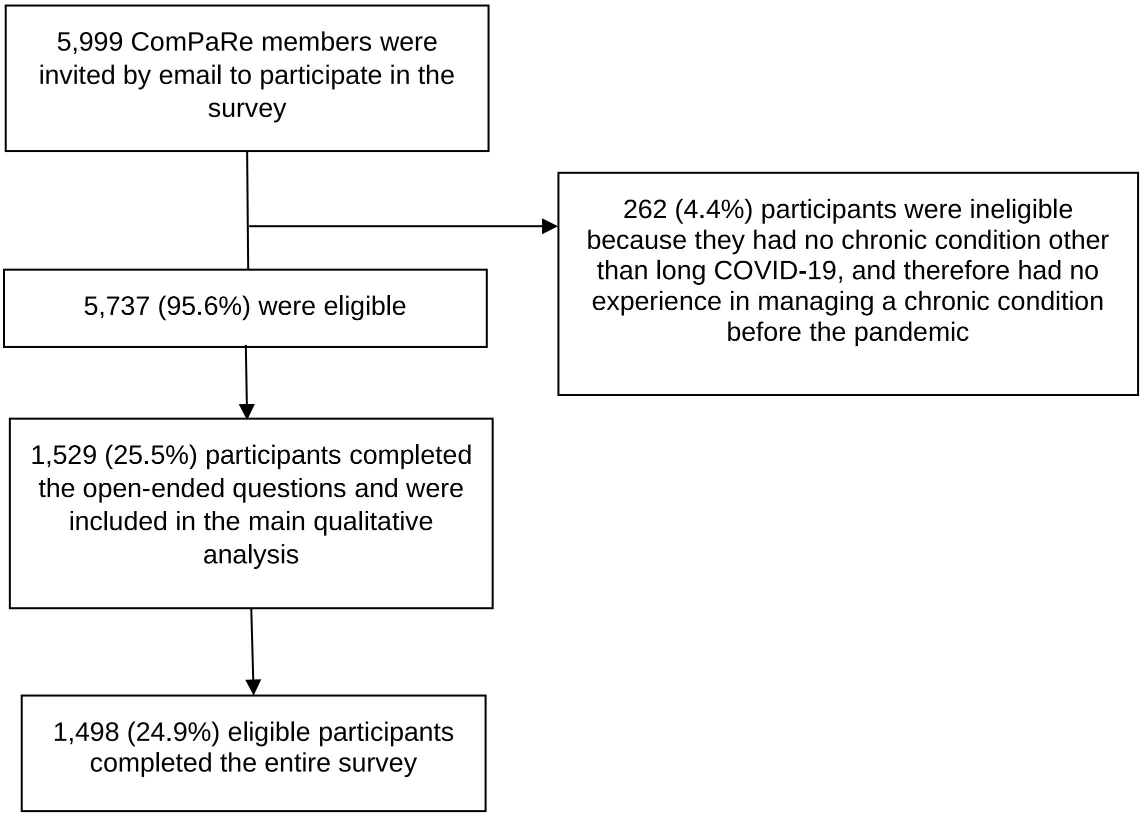

© 2021 Oikonomidi T et al. *JAMA Network Open.*

eFigure 2. Suggested uses of alternative care modalities implemented during the pandemic in post-pandemic care

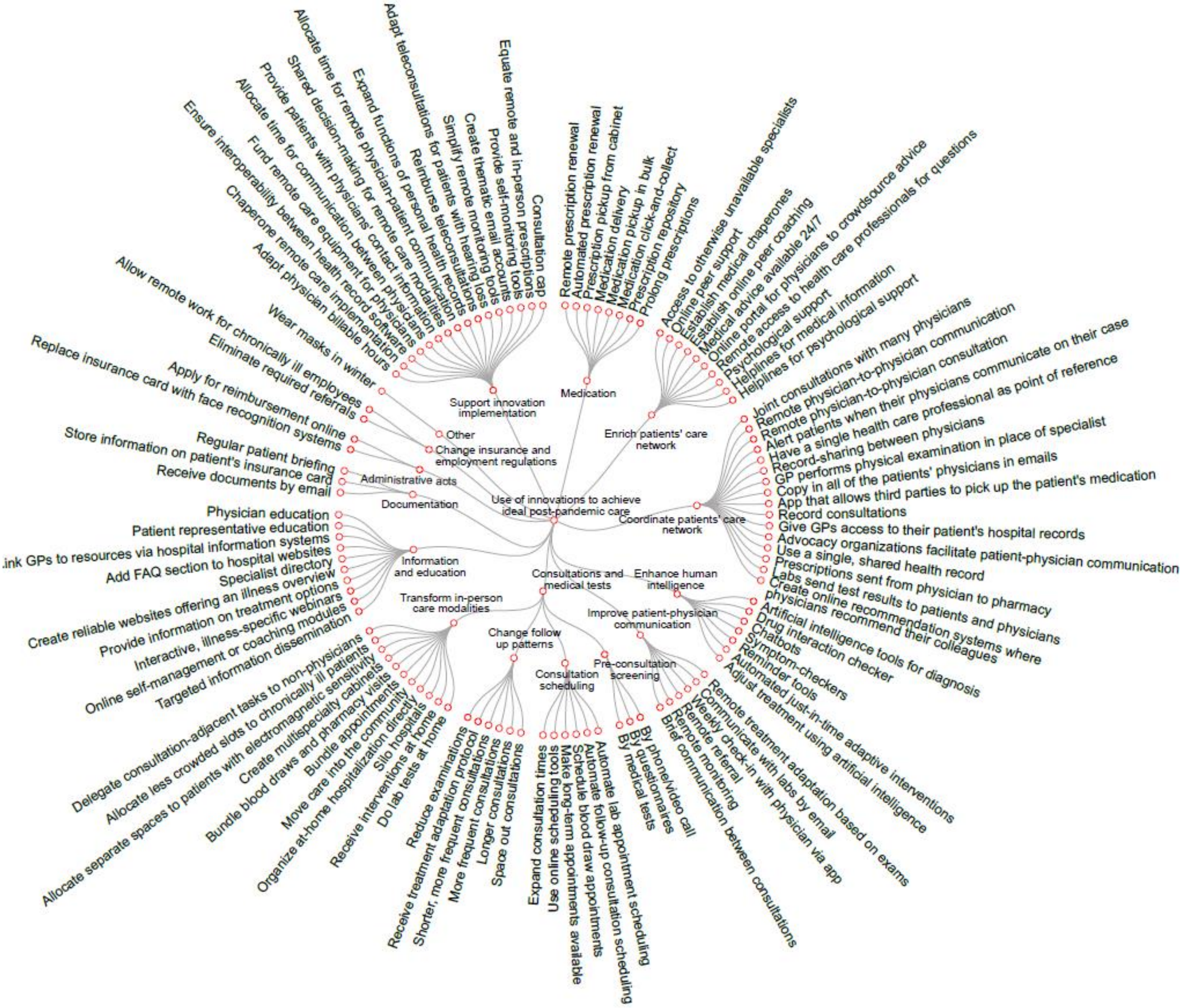

The figure presents 114 suggestions to use alternative care modalities to achieve patients' ideal post-pandemic care, as suggested by 1,529 study participants. The suggestions are grouped into 11 categories: consultations and medical tests (n=33 actions), improve patient-physician communication (n=6), medication (n=8), enrich patients' care network (n=10), coordinate patients' care network (n=16), enhance human intelligence (n=7), support blended care implementation (n=16), information and education (n=10), documentation (n=3), administrative acts (n=2), change insurance and employment regulations (n=2).

eFigure 3: Cumulative code accumulation curve representing data saturation

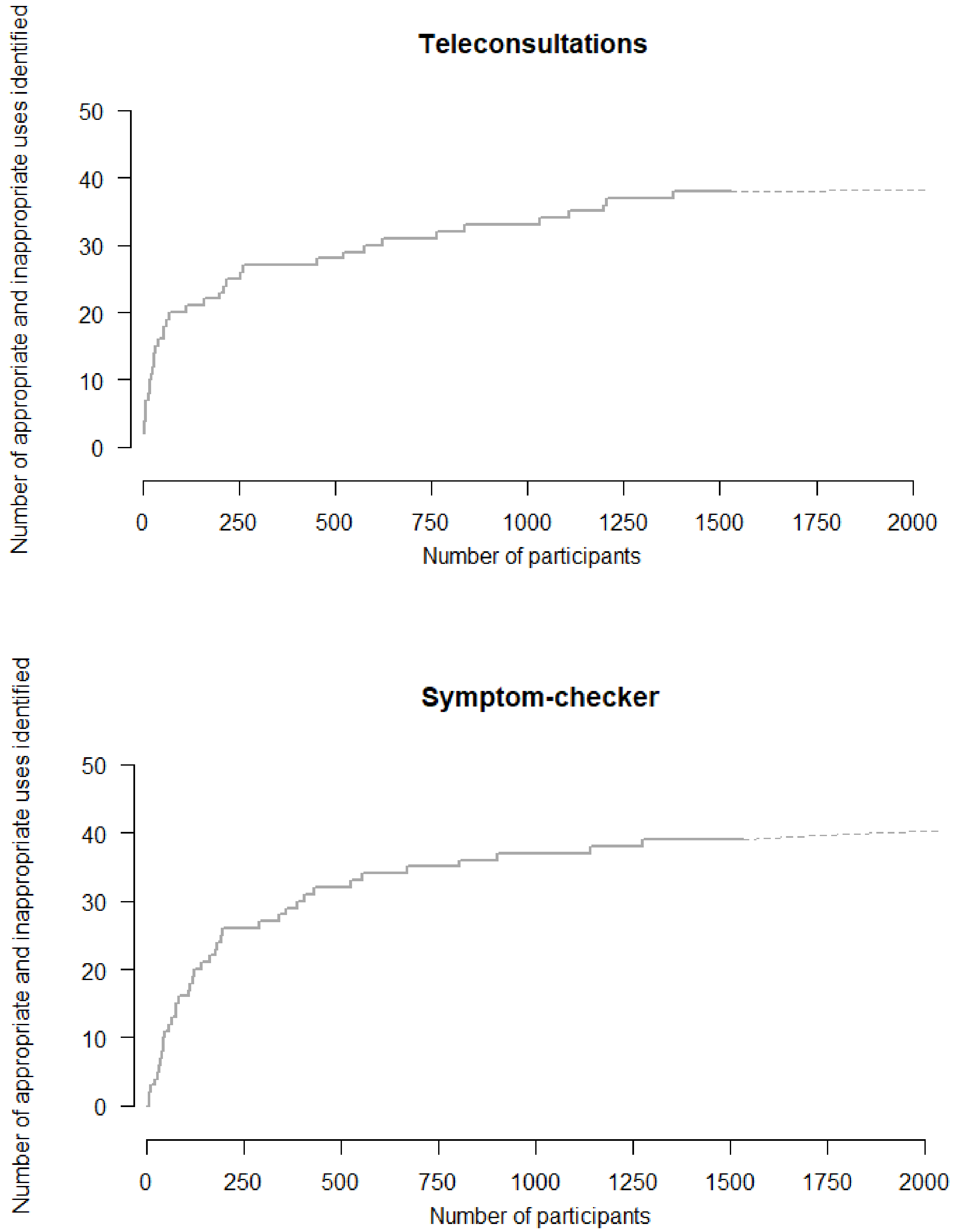

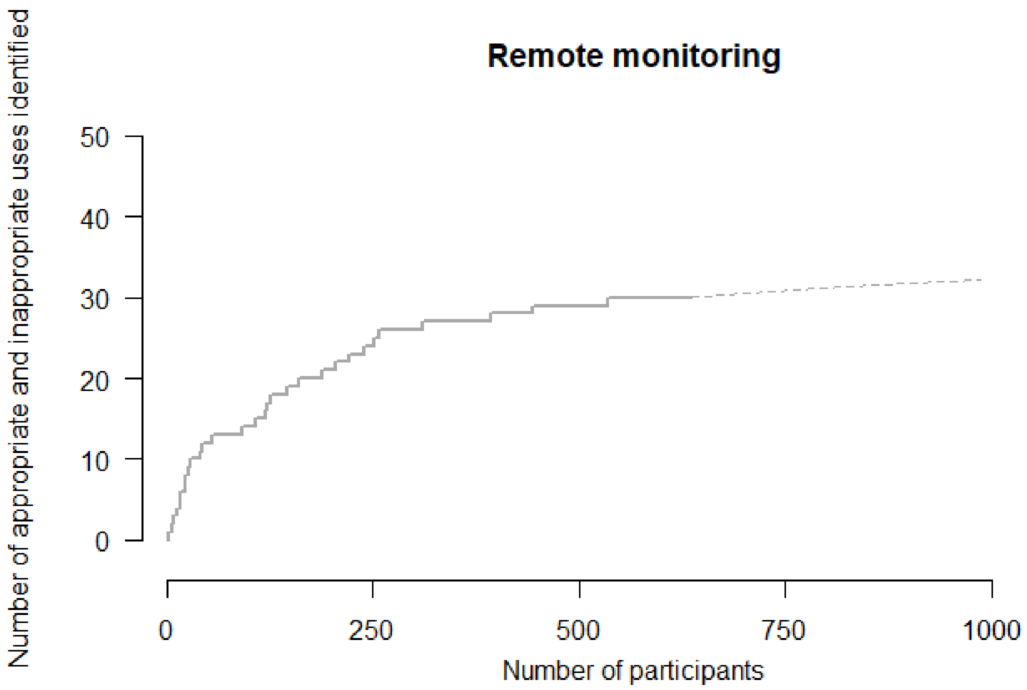

The continuous line in each panel represents the codes (i.e., appropriate and inappropriate uses of each alternative care modality) identified in the responses provided by 1,529 participants in our study. The dotted line represents the potential number of codes that could have been identified by recruiting 500 additional participants. Note that the sample size for remote monitoring is 636 instead of 1,529, because only participants who use monitoring to manage their illness were eligible to answer the question on remote monitoring.

eTable 1: Characteristics of responders, non-responders, and ComPaRe cohort participants (unweighted sample).

**Participant characteristics, N (%)**	**Non-responders (n=4208)**	**Responders (n=1529)**	**ComPaRe cohort (n=24374)** [f]
**Sex**			
Men	608 (20.4)	457 (29.9)	4605 (18.9)
Missing data	NA	NA	359 (1.5)
**Age (y), mean ± SD** [a]	44.13 ±14.13	50.28 ±14.73	43.79 (13.99)
**Education**			
Lower education	123 (2.9)	44 (2.9)	691 (2.8)
Middle school or equivalent	436 (10.4)	148 (9.7)	2568 (10.5)
High school or equivalent	717 (17.0)	226 (14.8)	4012 (16.5)
Associate's degree	811 (19.3)	323 (21.1)	4715 (19.3)
Undergraduate or graduate degree	2121 (50.4)	788 (51.5)	11216 (46.0)
Missing data	NA	NA	856 (35.1)
**Multimorbidity**			
Yes	2222 (52.8)	1062 (69.5)	10201 (41.9)
Missing data	NA	NA	358 (1.5)
**Years since first diagnosis, median [IQR]** [b]	10.00 [4.00, 22.00]	16.00 [6.00, 28.00]	9.00 [4.00, 21.00]
**Self-reported diagnosis** [c]			
Endometriosis	1357 (32.2)	303 (19.8)	8643 (35.5)
Depression	350 (8.3)	149 (9.7)	1458 (6.0)
High blood pressure	349 (8.3)	266 (17.4)	1764 (7.2)
Diabetes	264 (6.3)	148 (9.7)	1291 (5.3)
Cancer	222 (5.3)	114 (7.5)	1013 (4.2)
Asthma	300 (7.1)	130 (8.5)	1397 (5.7)
**Number of illnesses, median (interquartile range [IQR])** [d]	2.00 [1.00, 3.00]	2.00 [1.00, 4.00]	1.00 [1.00, 2.00]
**Total score, Treatment Burden Questionnaire, median (IQR)** [e]	53.00 [30.00, 79.00]	55.00 [29.00, 80.00]	NA

[a] Missing data for n=360 in the full ComPaRe cohort (last column).

[b] Missing data for n=359 in the full ComPaRe cohort (last column).

[c] Non-exhaustive list. List of the most frequently reported conditions. Some participants reported multiple conditions.

[d] Missing data for n=358 in the full ComPaRe cohort (last column).

[e] Missing data for n=1,358. This variable is not available for the entire ComPaRe cohort, because it is not part of the baseline questionnaire.

[f] Patients enrolled in the cohort that have provided informed consent and completed the baseline questionnaire. Data extracted on 27 January 2021.

© 2021 Oikonomidi T et al. *JAMA Network Open.*

eTable 2: Quantitative outcomes indicating the ideal use of alternative care modalities as a proportion of total relevant care needs, in the unweighted and weighted sample. [a]

**Outcome**	**Unweighted sample (n=1529)**	**Weighted sample (n=1529)**
**Consultations**		
Prefers primarily teleconsultations (alternative care modality)	476 (31.1)	477 (31.2)
Prefers primarily in-person consultations (traditional care modality)	752 (49.2)	719 (47.0)
Prefers entirely in-person consultations	277 (18.1)	312 (20.4)
No response	24 (1.6)	21 (1.3)
Ideal proportion of teleconsultations, median (interquartile range [IQR])	50.00 [20.00, 53.00]	50.00 [11.00, 52.00]
**Reacting to new symptoms**		
Prefers primarily to use symptom-checkers (alternative care modality)	314 (20.5)	357 (23.4)
Prefers primarily to contact their physician (traditional care modality)	702 (45.9)	574 (37.5)
Prefers entirely to contact their physician	482 (31.5)	564 (36.9)
No response	31 (2.0)	34 (2.2)
Ideal proportion of symptom-checker use, median [IQR]	22.50 [2.00, 50.00]	22.00 [2.00, 50.00]
**Monitoring [a]**	**n=636**	**n=669**
Prefers primarily remote monitoring and treatment adaptation (alternative care modality)	370 (58.1)	377 (56.4)
Prefers primarily to share monitoring data and adapt treatment during in-person consultations (traditional care modality)	197 (31.0)	192 (28.7)
Prefers entirely to share monitoring data and adapt treatment during in-personconsultations	69 (10.8)	100 (14.9)
No response	0 (0.0)	0 (0.0)
Ideal proportion of remote monitoring, median [IQR]	63.50 [40.00, 95.25]	52.30 [25.46, 85.41]

[a] Weighted data were obtained after calibration on margins for sex, age and educational level by using data from a national census describing the French population with chronic illness.

[b] Calculated only for participants who use monitoring to manage their illness. Participants who did not state they use monitoring were not eligible to answer this question.

eTable 3: Results of linear models in the weighted sample. [a,b]

	Ideal proportion of alternative care modality use								
	Teleconsultations (n=1299) [c]			Symptom-checker (n=1294)			Remote monitoring (n=544)		
**Predictors**	**Coefficient**	**95% CI**	**p**	**Coefficient**	**95% CI**	**p**	**Coefficient**	**95% CI**	**p**
(Intercept)	32.47	28.24 — 36.69	<0.001	32.83	28.72 — 36.94	<0.001	-3.26	-37.48 — 30.95	<0.001
**Participant characteristics**									
**Education (reference category: lower education)**									
Middle school or equivalent	-	-	-	-	-	-	32.04	12.81 — 51.26	0.001
High school or equivalent	-	-	-	-	-	-	32.03	13.79 — 50.27	<0.001
Associate’s degree	-	-	-	-	-	-	31.46	12.91 — 50.01	<0.001
Undergraduate or graduate degree	-	-	-	-	-	-	31.04	12.74 — 49.34	<0.001
**Feeling about household income (reference category: Finding it very difficult on present income)**									
Living comfortably on present income	-	-	-	-	-	-	40.92	9.93 — 71.91	0.009
**Endometriosis**	-	-	-	-8.45	-14.62 — -2.27	0.007	16.19	7.16 — 25.23	<0.001
**Cancer**	-	-	-	-	-	-	-27.08	-43.38 — -10.78	0.001
**Asthma**	-	-	-	-12.35	-21.86 — -2.83	0.011	-	-	-
**Prior teleconsultation use**	18.02	11.79 — 24.25	<0.001	-	-	-	-	-	-

[a] Weighted data were obtained after calibration on margins for sex, age and educational level by using data from a national census describing the French population with chronic illness.

© 2021 Oikonomidi T et al. *JAMA Network Open.*

[b] The models are fit in the complete-case subset (n=1299 for teleconsultations, n=1294 for symptom-checker, n=544 for remote monitoring).

[c] Adjusted R^2 for teleconsultation model: 0.09, for symptom-checker model: 0.017, for remote monitoring model: 0.24.

eTable 4: Code list for the appropriate and inappropriate uses of alternative care modalities. Overarching category labels in bold.

**Appropriate and inappropriate uses of alternative care modalities**							
**Code name**	**Code definition**	**Unweighted, teleconsultations (n=1529), n (%)**	**Weighted, teleconsultations (n=1529), n (%)**	**Unweighted, symptom-checker (n=1529), n (%)**	**Weighted, symptom-checker (n=1529), n (%)**	**Unweighted, remote monitoring (n=1529), n (%)**	**Weighted, remote monitoring (n=1529), n (%)**
**Care activities**							
Adapt treatment -	Remote care is inappropriate to confirm the efficacy of a newly prescribed treatment and quick treatment/dosage changes until the right fit for the patient is found.	24 (1.6)	10 (0.7)	0 (0.0)	0 (0.0)	0 (0.0)	0 (0.0)
Adapt treatment +	Remote care is appropriate to confirm the efficacy of a newly prescribed treatment and quick treatment/dosage changes until the right fit for the patient is found.	3 (0.2)	7 (0.4)	0 (0.0)	0 (0.0)	20 (3.1)	39 (5.8)
Address minor complaints +	Remote care is appropriate for minor complaints as opposed to serious, severe symptoms.	14 (0.9)	13 (0.8)	29 (1.9)	12 (0.8)	1 (0.2)	1 (0.2)
Address specific questions +	Remote care is appropriate to answer specific (i.e., limited in scope) questions of the patient or the physician.	37 (2.4)	17 (1.1)	4 (0.3)	8 (0.5)	0 (0.0)	0 (0.0)
Annual/in-depth consultation -	Remote care is inappropriate for the patient's annual consultation, the consultation in which the patient and the physician do a more in-depth review the patient's overall health status.	20 (1.3)	11 (0.7)	0 (0.0)	0 (0.0)	0 (0.0)	0 (0.0)
As a learning tool about the disease +	Remote care can be used to help patients understand their illness better.	0 (0.0)	0 (0.0)	1 (0.1)	6 (0.4)	1 (0.2)	1 (0.1)
As consultation aid +	The remote care tool can be used to collect information that can facilitate subsequent	0 (0.0)	0 (0.0)	24 (1.6)	12 (0.8)	7 (1.1)	6 (0.8)

	consultations with physicians (including to identify which physician they should contact, e.g., which of their specialists), or help patients better understand the diagnosis and instructions given to them by their physician after the consultation.						
Communication on sensitive issues -	Remote care is inappropriate for discussing sensitive topics, including receiving worrisome news (e.g., a new diagnosis).	10 (0.7)	4 (0.3)	1 (0.1)	0 (0.0)	0 (0.0)	0 (0.0)
Discuss medical test results -	Remote care is inappropriate for discussing medical test results.	2 (0.1)	12 (0.8)	0 (0.0)	0 (0.0)	0 (0.0)	0 (0.0)
Discuss medical test results +	Remote care is appropriate for discussing medical test results.	40 (2.6)	21 (1.3)	0 (0.0)	0 (0.0)	0 (0.0)	0 (0.0)
For informal caregivers +	The remote care tool can be used by the patient's informal caregivers to further support the patient.	0 (0.0)	0 (0.0)	2 (0.1)	2 (0.1)	0 (0.0)	0 (0.0)
For information purposes +	The remote care tool should be used for information purposes only, similar to a website publishing generic health information.	0 (0.0)	0 (0.0)	6 (0.4)	3 (0.2)	0 (0.0)	0 (0.0)
Identify symptom-disease link +	Remote care is appropriate to help patients identify if a new/worsening symptom is linked to their chronic disease or to a different disease.	0 (0.0)	0 (0.0)	9 (0.6)	7 (0.5)	1 (0.2)	0 (0.0)
Identify treatment misuse -	Remote care is inappropriate to identify the intentional or accidental misuse of potentially dangerous medication, such as opioids.	1 (0.1)	1 (0.0)	0 (0.0)	0 (0.0)	0 (0.0)	0 (0.0)
Identify treatment misuse +	Remote care is appropriate to identify the intentional or accidental misuse of potentially dangerous medication, such as opioids.	0 (0.0)	0 (0.0)	0 (0.0)	0 (0.0)	2 (0.3)	1 (0.1)
Joint consultations +	Remote care is appropriate for patients who would like to have consultations with more than one physician simultaneously.	3 (0.2)	1 (0.1)	0 (0.0)	0 (0.0)	0 (0.0)	0 (0.0)

© 2021 Oikonomidi T et al. *JAMA Network Open.*

Physical examination -	Remote care is inappropriate for physical examination. This includes any examination that traditionally requires the patient's physical presence, such as taking blood pressures, neurological tests, blood draw, ultrasounds, etc. Use this code if the participant says that in-person care is preferrable because it offers opportunity for physical-examination, which is necessary in medical care, or because their illness requires physical examination. Note that the need for physical examination does not have to be objective (e.g., participants may phrase it as a preference for physical examination). Do not use this code if the participant refers to the need or preference for in-person social contact with their physician without specifically referring to examination.	486 (31.8)	365 (23.9)	0 (0.0)	0 (0.0)	7 (1.1)	17 (2.5)
Physical intervention -	Teleconsultations are inappropriate when physical interventions, such as intravenous drips, are required.	35 (2.3)	30 (1.9)	0 (0.0)	0 (0.0)	0 (0.0)	0 (0.0)
Pluridisciplinary day hospital -	Remote care is inappropriate to replace the care received by multiple experts during day hospitalizations.	1 (0.1)	0 (0.0)	0 (0.0)	0 (0.0)	0 (0.0)	0 (0.0)
Prescription renewal +	Remote care is appropriate for renewing prescriptions.	209 (13.7)	188 (12.3)	0 (0.0)	0 (0.0)	2 (0.3)	7 (1.1)
Replace online information-seeking +	Remote care can be used to replace information-seeking in non-legitimate/unvetted websites or forums, which may give the patient erroneous information.	0 (0.0)	0 (0.0)	6 (0.4)	1 (0.1)	0 (0.0)	0 (0.0)
Routine follow-up consultations -	Teleconsultations are inappropriate for routine consultations (e.g., follow-up to control a stable condition, consultations	7 (0.5)	8 (0.5)	0 (0.0)	0 (0.0)	0 (0.0)	0 (0.0)

© 2021 Oikonomidi T et al. *JAMA Network Open.*

	described as "just a discussion to touch base" or "simple follow-ups" by participants). Do not use this code if the participants state a specific reason (corresponding to another code) why teleconsultations are inappropriate for routine follow-up, such as the need for physical examination.						
Routine follow-up consultations +	Teleconsultations are appropriate for routine consultations (e.g., follow-up to control a stable condition, consultations described as "just a discussion to touch base" or "simple follow-ups" by participants).	96 (6.3)	67 (4.4)	0 (0.0)	0 (0.0)	0 (0.0)	0 (0.0)
To prepare in-person consultation +	Teleconsultations are appropriate to decide if an in-person consultation is needed.	4 (0.3)	6 (0.4)	0 (0.0)	0 (0.0)	0 (0.0)	0 (0.0)
To rapidly appraise urgency +	Remote care is appropriate to appraise the urgency and severity one's symptoms and assess whether they should seek medical help. This helps patients feel reassured and avoid unnecessary consultations. Participants state that remote care can be used as a decision aid for the patient to estimate whether they should seek urgent care, schedule a consultation with their physician soon, or wait until the next scheduled consultation.	1 (0.1)	0 (0.0)	98 (6.4)	75 (4.9)	7 (1.1)	9 (1.3)
To supplement physician's abilities +	Remote care is appropriate when the patient's physician requires support (e.g., younger, less experienced physicians; physicians who have followed the patient for a long time and may become less attentive over time).	0 (0.0)	0 (0.0)	5 (0.3)	5 (0.4)	0 (0.0)	0 (0.0)

Urgent needs -	Remote care is inappropriate to address urgent needs in which medical advice is rapidly required.	1 (0.1)	0 (0.0)	12 (0.8)	27.3 (1.8)	0 (0.0)	0 (0.0)
Urgent needs +	Remote care is appropriate to address urgent needs in which medical advice is rapidly required. This may refer to participants' view that teleconsultation appointments are easier to obtain with shorter delays than in-person consultations, so that they can rapidly address emerging needs for medical care.	62 (4.2)	57 (3.7)	7 (0.5)	10 (0.6)	4 (0.6)	1 (0.2)
Use with General Practitioners (GPs) -	The remote care tool should not be used with one's general practitioner (as opposed to one's specialist).	0 (0.0)	0 (0.0)	0 (0.0)	0 (0.0)	1 (0.2)	0 (0.0)
Validate patient's self-diagnosis +	Remote care is appropriate for supporting patients' own expertise (e.g., after a patient examines their symptoms and decides on the best course of action, a symptom-checker can be used to confirm the patient's decision).	0 (0.0)	0 (0.0)	6 (0.4)	3 (0.2)	0 (0.0)	0 (0.0)
When other types of care are unavailable +	Using a symptom-checker is appropriate when traditional care is unavailable (e.g., on the weekend or at night, when the patient's physician cannot be reached, when the patient's physician may be unwilling to provide a phone consultation, when the next available consultation is too far from the appearance of a symptom).	0 (0.0)	0 (0.0)	30 (2.0)	35 (2.3)	1 (0.2)	0 (0.0)
**Care innovation characteristics**							
If data safety is guaranteed +	Data safety/online safety guarantees are a requisite (as a characteristic of the remote care tool) for appropriate remote care use	3 (0.2)	2 (0.1)	8 (0.5)	2 (0.1)	16 (2.5)	12 (1.9)

© 2021 Oikonomidi T et al. *JAMA Network Open.*

If it is used at the correct frequency +	Correct frequency of use is a requisite (as a characteristic of the remote care tool) for appropriate remote care use.	0 (0.0)	0 (0.0)	0 (0.0)	0 (0.0)	2 (0.3)	1 (0.1)
If it takes multimorbidity into account +	Remote care should be used if the tools take multimorbidity into account.	0 (0.0)	0 (0.0)	2 (0.1)	0 (0.0)	0 (0.0)	0 (0.0)
If payment/reimbursement is improved	Facilitation of the payment and reimbursement process is a requirement for the use of remote care.	2 (0.1)	7 (0.4)	0 (0.0)	0 (0.0)	0 (0.0)	0 (0.0)
If the algorithm is personalized +	A necessary condition to use symptom-checkers is that the patient's own data are included to complement the generic diagnostic algorithm.	0 (0.0)	0 (0.0)	8 (0.5)	3 (0.2)	0 (0.0)	0 (0.0)
If the measure is detailed enough +	Remote care should be used if the tools collect adequately detailed information.	0 (0.0)	0 (0.0)	0 (0.0)	0 (0.0)	2 (0.3)	0 (0.1)
If the patient has control over sending the data +	Remote care should be used if the patient has control over when their data will be sent to or accessed by their physician.	0 (0.0)	0 (0.0)	0 (0.0)	0 (0.0)	4 (0.6)	2 (0.2)
If the tool is explained by the physician +	A necessary condition to use symptom-checkers is that the physician explains their use to the patient in advance.	0 (0.0)	0 (0.0)	2 (0.1)	1 (0.1)	2 (0.3)	1 (0.1)
If the tool is supervised by a physician +	Use of remote care tools (symptom-checkers and remote monitoring) requires that they are supervised by a physician in one of the following ways: 1) in case of alert, the results of the symptom-checker are directly sent to a physician who takes over the process, or 2) when the symptom-checker has produced a result, a physician calls the user to review/go in-depth in the diagnostic (regardless of alert), 3) using remote monitoring requires the certainty that the monitored data will be reviewed and taken into account by the physician.	0 (0.0)	0 (0.0)	13 (0.9)	4 (0.3)	23 (3.6)	27 (4.1)

If the tools are provided to patients +	Being provided with the monitoring tools is necessary for the patient to take up remote monitoring.	0 (0.0)	0 (0.0)	0 (0.0)	0 (0.0)	4 (0.6)	2 (0.3)
If there is quality assurance +	Quality assurance or vetting (such as an official recommendation of a symptom-checker website from the French national health system) is a requisite (as a characteristic of the remote care tool) for appropriate remote care use, to separate legitimate from lesser-quality versions of the remote care tool. Participants state they would need help to judge if a tool is trustworthy in order to use it.	0 (0.0)	0 (0.0)	27 (1.8)	12 (0.8)	2 (0.3)	1 (0.1)
**Patient characteristics**							
Emerging illnesses -	Remote care is appropriate for illnesses known in the medical community. It is inappropriate for new, emerging illnesses for which a solid knowledge base does not exist.	1 (0.1)	0 (0.0)	2 (0.1)	1 (0.0)	0 (0.0)	0 (0.0)
Employed patients +	Remote care is appropriate for employed patients who may find it difficult or undesirable to take time off work for in-person care.	19 (1.2)	12 (0.8)	1 (0.1)	0 (0.0)	0 (0.0)	0 (0.0)
Established patient-physician relationship +	Remote care should be used only after a supportive and trusting patient-physician relationship has been established.	10 (0.7)	8 (0.5)	0 (0.0)	0 (0.0)	5 (0.8)	1 (0.2)
Heterogeneous, multifactorial or atypical symptoms -	Remote care is inappropriate for patients whose symptoms are atypical for their condition, or symptoms that vary in their manifestation from day to day, and are affected by multiple factors (e.g., stress, nutrition, etc.)	0 (0.0)	0 (0.0)	19 (1.2)	8 (0.5)	1 (0.2)	0 (0.0)
Infrequent consultations -	Remote care is inappropriate for patients who already rarely consult their physician	24 (1.6)	11 (0.7)	1 (0.1)	0.3 (0.0)	0 (0.0)	0 (0.0)

© 2021 Oikonomidi T et al. *JAMA Network Open.*

	in person. This refers to the participant's subjective appraisal of consultations as infrequent, regardless of the actual frequency.						
Infrequent consultations +	Remote car is appropriate for patients who rarely consult their physician in person. This refers to the participant's subjective appraisal of consultations as infrequent, regardless of the actual frequency.	0 (0.0)	0 (0.0)	0 (0.0)	0 (0.0)	6 (0.9)	4 (0.6)
Initial consultations -	Remote care is inappropriate for initial consultations establishing a new diagnosis and for newly diagnosed patients.	10 (0.7)	6 (0.4)	0 (0.0)	0 (0.0)	0 (0.0)	0 (0.0)
Initial consultations +	Remote care is appropriate for initial consultations establishing a new diagnosis and for newly diagnosed patients.	0 (0.0)	0 (0.0)	3 (0.2)	1 (0.1)	0 (0.0)	0 (0.0)
Lack of private personal space -	Teleconsultations are inappropriate for patients who do not have a private space at home where they can speak to their physician uninterrupted and in confidentiality (e.g., because they live with family).	6 (0.4)	4 (0.2)	0 (0.0)	0 (0.0)	0 (0.0)	0 (0.0)
Limited treatment variation -	Remote care is inappropriate for patients whose treatment protocol is rarely modified/cannot be modified irrespective of their symptoms, as opposed to patients whose treatment depends on their symptoms or vitals.	0 (0.0)	0 (0.0)	0 (0.0)	0 (0.0)	4 (0.6)	2 (0.3)
Areas with few available physicians +	Remote care is appropriate for patients living in the countryside/locations with few available physicians in geographic proximity or far from reference centers specializing in the patient's illness.	48 (3.1)	51 (3.3)	3 (0.2)	19 (1.2)	0 (0.0)	0 (0.0)
Multimorbid patients -	Remote care is inappropriate for patients with multimorbidity (e.g., because of the burdensome nature of their care).	0 (0.0)	0 (0.0)	3 (0.2)	0 (0.0)	0 (0.0)	0 (0.0)

© 2021 Oikonomidi T et al. *JAMA Network Open.*

Multimorbid patients +	Remote care is appropriate for patients with multimorbidity (e.g., because of the burdensome nature of their care).	2 (0.1)	1 (0.0)	1 (0.1)	0 (0.0)	0 (0.0)	0 (0.0)
Patient expertise -	Remote care is inappropriate for patients who have in-depth knowledge and experience managing their illness, because it has little to contribute.	0 (0.0)	0 (0.0)	27 (1.8)	11 (0.7)	5 (0.8)	2 (0.2)
Patient expertise +	Remote care is appropriate for patients who have in-depth knowledge and experience managing their illness. Knowledge and experience are prerequisites that enable patients to use the tool efficiently and correctly.	4 (0.3)	9.1 (0.6)	10 (0.7)	3 (0.2)	5 (0.8)	4 (0.5)
Patient self-monitors +	For remote monitoring: it is appropriate for patients who already have a habit of self-monitoring. For teleconsultations: they are appropriate for patients who can self-monitor to enrich teleconsultations by providing monitoring data (e.g., blood pressure readings) to skip physical examinations.	10 (0.7)	11 (0.7)	0 (0.0)	0 (0.0)	4 (0.6)	2 (0.2)
Patients prone to anxiety regarding their health -	Symptom-checkers are inappropriate for patients who are likely to experience anxiety about their health. Use of the tool may lead these patients to overestimate the gravity of their symptoms or encourage rumination on their symptoms.	0 (0.0)	0 (0.0)	45 (2.9)	35 (2.3)	0 (0.0)	0 (0.0)
Patients requiring closer follow-up than that offered by traditional care +	Remote care is appropriate for patients who require or prefer closer follow-up than that offered by traditional care in which consultations are infrequent, so that their treatment can be adapted quickly after a change in the patient's condition.	0 (0.0)	0 (0.0)	0 (0.0)	0 (0.0)	36 (5.7)	59 (8.8)

© 2021 Oikonomidi T et al. *JAMA Network Open.*

Patients who are temporarily away from home +	Remote care is appropriate for when patients are temporarily away from home on vacation or business, so that their care is not interrupted by their absence.	8 (0.5)	15 (1.0)	0 (0.0)	0 (0.0)	0 (0.0)	0 (0.0)
Patients with limited remote communication capacity -	Remote care is inappropriate for patients who have trouble with remote communications due to their illness (e.g., telephone phobia).	4 (0.3)	1 (0.1)	0 (0.0)	0 (0.0)	0 (0.0)	0 (0.0)
Patients with restricted mobility +	Remote care is appropriate for patients with reduced mobility due to physical or mental illness. Includes patients who experience physical pain when seated at waiting rooms for a long time.	32 (2.1)	37 (2.4)	1 (0.1)	0 (0.0)	0 (0.0)	0 (0.0)
Stable illness -	Remote care is inappropriate for patients with controlled, stable illness (i.e., in the absence of new symptoms or other evolution of the illness).	0 (0.0)	0 (0.0)	4 (0.3)	2 (0.1)	9 (1.4)	8 (1.1)
Stable illness +	Remote care is appropriate for patients with controlled, stable illness (i.e., in the absence of new symptoms or other evolution of the illness.	124 (8.1)	126 (8.3)	5 (0.3)	7 (0.4)	7 (1.1)	3 (0.5)
Symptoms can be observed and reported +	A necessary condition to use symptom-checkers is that the symptoms can be observed and reported accurately by patients with no need for special training. This depends on the illness (e.g., certain illnesses may be asymptomatic, or the symptoms may be difficult to accurately observe without help from a physician, such as psychiatric or dermatologic illnesses).	0 (0.0)	0 (0.0)	43 (2.8)	28 (1.8)	4 (0.6)	2 (0.3)
To keep physician after moving +	Remote care is appropriate for patients who wish to maintain their physician after moving to a new location.	1 (0.1)	0 (0.0)	0 (0.0)	0 (0.0)	0 (0.0)	0 (0.0)

© 2021 Oikonomidi T et al. *JAMA Network Open.*

[a] Weighted data were obtained after calibration on margins for sex, age and educational level by using data from a national census describing the French population with chronic illness.

eTable 5: Code list for the attributes of ideal care and suggested uses of alternative care modalities to achieve ideal care. Overarching category labels in bold.

**Attributes of ideal care**			
**Code name**	**Code definition**	**Unweighted (n=1529), n (%)**	**Weighted (n=1529), n (%)**
Responsive	Care will be more dynamic and responsive to patients' needs. Patients will receive care (e.g., consultations with a specialist, treatment adaptation) exactly when care is needed, without having to wait several months for a consultation. Consultations will be responsive to patients' needs instead of following a non-personalized template that mandates follow-up appointments at pre-specified time intervals.	270 (17.7)	206 (13.5)
Empathetic	Care will be characterized by empathetic communication between patients and physicians. Physicians will have good communication skills and patients will feel heard, seen and understood.	77 (5.0)	63 (4.1)
Interconnected	The professionals involved in the patient's care network will communicate with each other to facilitate information exchange. The patient will not have to be the sole messenger transferring information on their illness(es) between professionals.	139 (9.1)	90 (5.9)
Collective	Care is described as a system in which resources are shared (e.g., less severely ill patients will receive remote care to free physicians' time for more severely ill patients).	10 (0.7)	3 (0.2)
Safe from infectious diseases	Refers to care being safe from infectious diseases (e.g., annual flu).	31 (2.0)	45 (2.9)
Holistic	Care will be holistic, treating the patient as a person instead of treating individual organs. This includes offering psychological support to patients.	58 (3.8)	29 (1.9)
Less burdensome to informal caregivers	Care will be less burdensome to patients' informal caregivers (i.e., family and friends who help the patient with their care).	6 (0.4)	4 (0.3)
Autonomy-supportive	Care will give patients more autonomy in the management of their health.	23 (1.5)	12 (0.8)
Single-tier	Blended care will not be a two-tier system, i.e., of higher quality for patients who can use remote technologies, and of lesser quality for patients who cannot.	22 (1.4)	31 (2.0)
Physician availability	Refers to the need for a greater number of physicians in general, or of physicians that are competent at managing the patient's illness. This code was not used for quotes that refer specifically to the time it takes to get a consultation with a specialist (the code Reactive was used instead).	62 (4.1)	43 (2.8)
Minimally disruptive	Care will disrupt patients' personal lives less (e.g., having to take time off work to attend in-person consultations).	52 (3.4)	20 (1.3)

© 2021 Oikonomidi T et al. *JAMA Network Open.*

More affordable	Out-of-pocket costs associated with care will be reduced.	46 (3.0)	36 (2.4)
Lean	There will be fewer in-person consultations considered unnecessary by the patient, thereby reducing associated travel and wait, the term "less heavy" was also coded as "Lean".	470 (30.7)	432 (28.2)
More personalised	Care will be personalised to each individual patient.	27 (1.8)	16 (1.0)
Informative	Information about the patients' illness and care will be clearly provided during their care, from official sources, such as by their physician, including orientation towards the right specialist for their illness. Patients will not have to struggle to get information about their illness or resort to unverified online resources. This refers to information about the illness and treatments, not one's personal data (e.g., adding exam results to a patient's health records).	45 (2.9)	34 (2.2)
Not redundant	Care will not require patients to do the same thing multiple times (e.g., to do the same blood test twice, because it was ordered by two different specialists a few weeks apart).	16 (1.0)	10 (0.7)
Automated	Some processes of care should be automated, instead of requiring vigilance and action from patients (e.g., taking follow-up appointments, having prescriptions automatically renewed without having to put in a request).	17 (1.1)	10 (0.7)
Closer follow-up	Patients will be followed more closely and regularly, by having more consultations, by having more frequent contact with their physician in-between consultations, or by using remote monitoring.	64 (4.2)	60 (3.9)
Better documented	The patient's care will be better documented. For example, all documents regarding the patient's illness, such as lab test results, will be available to patients and stored in their medical record, patients will regularly receive reports providing an overview of their illness and consultation 'minutes'. This is associated with reducing redundancy and improving information flows within the patient's care network (see codes "Not redundant" and "Interconnected"). This code does not refer to generic information about the patient's illness, but to their documentation of data produced by their personal care.	35 (2.3)	21 (1.4)
Maintain in-person patient-physician contact	Care will be at least partially based on in-person patient-physician encounters. It will not be fully remote, either for practical reasons (e.g., need for physical examination) or for social reasons (e.g., to maintain human contact and facilitate the patient-physician relationship) or due to patient preferences and beliefs regarding the superiority of in-person care.	233 (15.2)	199 (13.0)
As before	Care should return to traditional, pre-pandemic care. No remote care modalities used during the pandemic should be adopted in the long term, either because the patient's pre-pandemic care is ideal, or because they do not believe that their care can change drastically (e.g., because they consider pre pandemic levels of in-person care to be necessary).	131 (8.6)	143 (9.4)
Linked to research	Data collected in real-life settings, such as by self-monitoring, will be used in research to advance knowledge on the patient's illness.	1 (0.1)	0 (0.0)

© 2021 Oikonomidi T et al. *JAMA Network Open.*

**Suggested use of alternative care modalities to achieve ideal care**			
**Code name**	**Code definition**	**Prevalence in unweighted dataset, n (%)**	**Prevalence in weighted dataset, n (%)**
**Consultations and medical tests**	**Modifications to the scheduling or implementation of consultations or medical tests.**		
Consultation scheduling			
Automate lab appointment scheduling	When patients are prescribed lab tests or other examinations (e.g., ultrasound), there should be an automated process of taking an appointment for the tests.	1 (0.1)	0 (0.0)
Automate follow-up consultation scheduling	Pre-schedule the follow-up consultation (when needed) during the current consultation.	9 (0.6)	8 (0.5)
Schedule blood draw appointments	Patients will be able to book blood draw appointments with labs to avoid wait.	1 (0.1)	0 (0.0)
Make long-term appointments available	Patients will be able to make appointments further into the future.	1 (0.1)	0 (0.0)
Use online scheduling tools	Consultations (including hospital consultations) will be scheduled online via a dedicated website. The consultation scheduling website will offer functions such as presenting all available time slots so that the patient can choose the most convenient time slot, alerts when earlier consultation slots become available due to cancellation, and the option to select the reason for consultation (e.g., renew prescription, adapt treatment due to side effects), offering different time slots depending on urgency.	77 (5.0)	54 (3.5)
Expand consultation times	Expand the time slots available for patients to consult their physicians.	2 (0.1)	0 (0.0)
Pre-consultation screening			
Pre-consultation screening (phone/video call)	Patients have a brief phone or video call with their physician to decide if they need a consultation.	5 (0.3)	2 (0.2)
Pre-consultation screening (questionnaires)	Have a screening system to decide if the patient needs a consultation based on responses to regularly completed questionnaires on their symptoms.	3 (0.2)	1 (0.0)

Pre-consultation screening (medical tests)	Have a screening system to decide if the patient needs a consultation based on lab test results.	6 (0.4)	2 (0.1)
Change follow-up patterns			
Space out consultations	Prolong the time between consultations.	26 (1.7)	15 (1.0)
Longer consultations	Give patients more time with their physician per consultation	10 (0.7)	5 (0.3)
More frequent consultations	Patients will be able to consult their physician more frequently, in part thanks to remote care modalities.	19 (1.2)	16 (1.1)
Shorter, more frequent consultations	Care should comprise shorter but more frequent consultations.	5 (0.3)	1 (0.1)
Receive treatment adaptation protocol	Patients receive a protocol guiding them to test different treatments (e.g., different doses of the prescribed medication) until they find the one they respond best to.	1 (0.1)	0 (0.0)
Reduce examinations	Reduce the number of medical examinations and tests requested of patients.	1 (0.1)	1 (0.0)
Transform in-person care modalities			
Do lab tests at home	Biological samples required for lab tests, such as blood draws, will be taken at home by a health care professional or by the patient and then sent to a lab for analysis.	10 (0.7)	4 (0.3)
Receive interventions at home	Deliver interventions that are usually done in hospital, such as IV drips, at home instead.	11 (0.7)	14(0.9)
Silo hospitals	Establish separate hospitals for different patient groups according to their illness.	1 (0.1)	1 (0.0)
Organize at-home hospitalization directly	The patient can benefit from at-home hospitalization without having to pass by the emergency room first.	1 (0.1)	0 (0.0)
Move care into the community	Patients will be able to do medical acts, such as lab tests and medical visits, in their local community, instead of having to go to the hospital. For example, specialist physicians could practice at community health centres on prespecified dates, instead of patients having to travel to medical centres specialized in their illness located in a different region.	9 (0.6)	11 (0.7)
Bundle appointments	Bundle different appointments (exams, consultations) at the same place, on the same day	17 (1.1)	16 (1.0)
Bundle blood draws and pharmacy visits	Offer the option to do blood draws at the pharmacy and directly pick up the adapted medication based on the results.	1 (0.1)	1 (0.1)
Create multispecialty offices	Create medical offices that group physicians of all specialties relevant to a specific illness, including medical laboratory services, as a "one stop shop" for patients with a given illness.	5 (0.3)	2 (0.1)
Allocate separate spaces to patients with	Provide adapted care spaces for patients with Multiple chemical sensitivity and Electromagnetic hypersensitivity.	1 (0.1)	0 (0.0)

© 2021 Oikonomidi T et al. *JAMA Network Open.*

electromagnetic or multiple chemical sensitivity			
Allocate less crowded slots to chronically ill patients	Allocate less crowded time slots to chronically ill patients for in-person consultations, so they come in contact with fewer people (e.g., to minimize the risk of contracting an infectious disease)	1 (0.1)	0 (0.0)
Delegate consultation-adjacent tasks to non-physicians	Some consultation-adjacent tasks could be done by trained nurses or pharmacists. These may include routine consultations (in which the main aim is to check that the patient's condition remains stable by means of physical examination or review of test results, and renew their prescription), consultations aiming at prevention, or consultations with specialized nurses who, either alone or by consulting with a specialized physician, can support the patient and their family physician in managing the illness. Physicians could also collaborate with nurses in delivering blended care (e.g., local nurses could perform a physical examination and take the patient's vitals and transmit the data to the patient's physician, who then can adapt the patient's medication if needed).	8 (0.5)	11 (0.7)
Introduce remote care modalities			
Use teleconsultations	Participants suggested that teleconsultations should be part of post-pandemic care. Participants differ regarding the way in which they suggest teleconsultations be used. Some participants emphasized that the choice of teleconsultations versus in-person consultations should be left up to the patient, and others highlighted the need to keep some in-person consultations. Some participants suggested that teleconsultations be used under specific conditions (e.g., when the illness is stable and no physical exam is required). Finally, some participants reported that teleconsultations would result in more regular or frequent care, and that teleconsultation appointments are easier to get with shorter delays, compared to in-person consultations.	658 (43.0)	594 (38.9)
Online physiotherapy	Replace physiotherapy consultations with pre-recorded or live online physiotherapy sessions.	2 (0.1)	0 (0.0)
Consultation-preparatory questionnaires	Before each consultation, patients can fill in questionnaires to provide physicians with information about their health.	1 (0.1)	0 (0.0)
Remote prescription for lab tests before consultation	Patients will be able to remotely receive prescriptions for lab tests (e.g., via email or through the patient portal associated with their personal health record). This will save patients a consultation whose sole purpose is the prescription of lab tests, and it can help patients do lab tests rapidly in response to the evolution of their illness (e.g., as soon as their symptoms worsen).	21 (1.4)	14 (0.9)
Text-based consultations	Patients will have the option of doing consultations in writing (by email, chat or text messaging), without verbal communication via video or phone calls, potentially supplemented by data (e.g., photographs of affected areas).	3 (0.2)	1 (0.1)

Joint consultations with many patients	Organize consultations where many patients can simultaneously consult with the same physician, on rather general, non-confidential topics.	2 (0.1)	1 (0.1)
Test patient-physician fit using teleconsultation	When patients seek a new physician, they can have a first teleconsultation to decide if they would like to pursue a therapeutic relationship with this physician.	3 (0.2)	1 (0.1)
**Improve patient-physician communication**	**Make communication between the patient and their regular physician(s) more responsive to the patient's needs outside of consultations.**		
Remote treatment adaptation based on exams	The patient's lab test results are sent to their physician, who then remotely adapts the patient's treatment, or simply renews their prescription remotely, without consultation. Additional information can be provided, such as the results of physical exams performed by non-physician health care professionals.	5 (0.3)	7 (0.5)
Communicate with labs by email	Patients will be able to communicate with biomedical labs by email.	1 (0.1)	0 (0.0)
Weekly check-in with physician via app	Patients will be able to communicate with their regular physicians once a week via a dedicated app.	1 (0.1)	0 (0.0)
Remote referral	Obtain referral letter to a specialist physician remotely.	2 (0.1)	1 (0.1)
Brief communication between consultations	Being able to contact one's regular physician briefly, to address specific concerns or questions (e.g., about side effects of prescribed medication) in a synchronous or asynchronous manner. This may take the form of "mini teleconsultations" (i.e., brief 5- or 10-minute contact by phone or video call to address specific topics between proper consultations), e-mails, or chat via messaging platforms.	222 (14.5)	135 (8.8)
Remote monitoring	Transmission of monitoring data from a monitoring tool, such as a wearable sensor, a medical device, or a symptom log, to one's physician, for reactive treatment adaptation	92 (6.0)	66 (4.3)
**Medication**	**Facilitate patients' access to medication.**		
Remote prescription renewal	Patients will be able to obtain a prescription remotely, such as by email, after teleconsultations. This includes prescriptions for medication and lab tests.	322 (21.1)	283 (18.5)
Automated prescription renewal	The patient will have their medication prescription renewed remotely, without having a consultation or teleconsultation. The renewal may be automated for the same prescription, if the patient does not notify the physician of change in their condition, or it may be coupled with remote monitoring (i.e., the physician can renew or send a modified prescription, depending on the patient's monitoring data).	52 (3.4)	43 (2.8)
Prescription pickup from medical offices	The patient's prescription is renewed by the physician and the patient picks it up at their medical office/from their secretary without having to wait.	2 (0.1)	0 (0.0)
Medication delivery	Medication will be delivered to the patient's home. For patients who pick up their medication from the hospital pharmacy, medication may be delivered to their local pharmacy for pickup.	79 (5.2)	60 (3.9)

© 2021 Oikonomidi T et al. *JAMA Network Open.*

Medication pickup in bulk	Being able to pick up medication for more than 1 month at each pharmacy visit.	16 (1.0)	16 (1.0)
Medication click-and-collect	Patients can pre-order their medication and pick it up at their local pharmacy. This saves patients having to wait for their order to be put together at the pharmacy and spares them unnecessary trips to their pharmacy when their medication is out of stock.	9 (0.6)	5 (0.3)
Prescription repository	Online repository where physicians upload prescriptions for patients to download (possibly in a format that can be easily shared with their pharmacist, such as a QR code).	3 (0.2)	2 (0.2)
Prolong prescriptions	Prolong prescription validity	19 (1.2)	10 (0.6)
**Enrich patients' care network**	**Multiply the nodes in the patient's care network.**		
Access to otherwise unavailable specialists	Patients can use communication technologies to remotely consult a specialist in their illness other than their regular physician. Technologies enable patients to reach experts that would otherwise be inaccessible because of distance, or to reach many experts to efficiently obtain several opinions.	13 (0.9)	12.6 (0.8)
Online peer support	Online support groups by peers with the same illness.	8 (0.5)	3.1 (0.2)
Establish "medical chaperones"	Create 'medical chaperones': non-physician professionals charged with supporting the care of patients, where physician skills are not required (e.g., informing patients about annual vaccination campaigns, teaching patients healthy cooking skills via online classes).	1 (0.1)	0.8 (0.1)
Establish online peer coaching	Establish an online platform where trained peers with the same illness as the patient provide support, supervised by a physician.	1 (0.1)	0 (0.0)
Medical advice available 24/7	Patients will be able to receive medical advice around the clock.	7 (0.5)	11.4 (0.7)
Online portal for physicians to crowdsource advice	Create a secure online portal for physicians to obtain colleagues' advice and opinions on a specific patient, identified through their national health insurance number.	1 (0.1)	0.4 (0.0)
Remote access to health care professionals for questions	Give patients the possibility to remotely contact health care professionals other than their own physician, to ask questions and receive responses in real time. This may take the form of a website, direct chat, or videocall.	5 (0.3)	8.9 (0.6)
Psychological support	Provision of psychological support to patients.	14 (0.9)	10.3 (0.7)
Helplines for medical information	Create helplines that offer information to patients regarding their illness, including answers to specific, urgent questions.	16 (1.0)	13.1 (0.9)
Helplines for psychological support	Create helplines that offer psychological support to patients. The hotlines may be staffed by health care professionals or trained peers with the same illness as the patient calling.	20 (1.3)	23.9 (1.6)
**Coordinate patients' care network**	**Close the edges between unconnected nodes in the patient's care network.**		

© 2021 Oikonomidi T et al. *JAMA Network Open.*

Joint consultations with many physicians	Patients will be able to have consultations with more than one physician simultaneously (e.g., GPs and different specialists).	13 (0.9)	5 (0.3)
Remote physician-to-physician communication	The patient's physicians will communicate with each other. For example, physicians of different specialties may contact each other to coordinate regarding the medications prescribed to the patient (to examine counter-indications due to one of the patient's illnesses, possible medication interactions, etc.), or they may exchange the results of examinations, lab tests and imaging. This does not refer to joint consultations in which the patient consults with multiple physicians simultaneously, but to communication between physicians in which the patient is not present. Communication also differs from physician-to-physician consultation, in that it refers to the bidirectional exchange of information about the patient, and not to a physician consulting another for a specialist opinion.	58 (3.8)	25 (1.6)
Remote physician-to-physician consultation	The patient's physician will consult with a specialist regarding the patient's case, either to spare the patient an additional consultation with a specialist, or because the specialist may be otherwise inaccessible to the patient (e.g., consulting with an international expert on the patient's condition). The physician may contact the specialist via an online platform and share patient data with consent (e.g., test results, the recording of the physical examination or the entire consultation between the patient and their own physician).	35 (2.3)	25 (1.6)
Alert patients when their physicians communicate on their case	When physicians communicate on their common patient, the patient will receive an alert.	1 (0.1)	0 (0.0)
Have a single health care professional as "point of reference"	Patients will have a specific, single health care professional (most often proposed to be the family physician, but also potentially a specialized physician or a nurse) who will manage and coordinate the care of their chronic illness(es), consult other physicians on behalf of the patient if necessary, and chaperone patients in solving any problems that may come up (e.g., in their interaction with medical laboratories).	20 (1.3)	13 (0.8)
Record-sharing between physicians	Physicians will share their records of their common patients.	2 (0.1)	5 (0.3)
GP performs physical examination in place of specialist	If the patient's specialist is difficult to access (e.g., because they are located in a different city), physical examinations can be performed by the patient's GP and communicated to their specialist, to facilitate teleconsultations with the specialist.	1 (0.1)	0 (0.0)
Copy all of the patients' physicians in emails	When a physician communicates with their patient by email, or when a lab sends patients their test results, all of the patient's physicians should be copied.	2 (0.1)	1 (0.1)
App that allows third parties to pick up the patient's medication	Create an app that allows third parties to pick up the patient's medication	1 (0.1)	1 (0.1)

© 2021 Oikonomidi T et al. *JAMA Network Open.*

Create online recommendation systems where physicians recommend their colleagues	Create online recommendation systems where patients can find more physicians that have been recommended by their current physicians	1 (0.1)	1 (0.1)
Record consultations	Be able to record consultations. The recording can be used to remind patients the information discussed in the consultation or it can be shared with another physician who can offer a specialized opinion on the patient's case.	2 (0.1)	0 (0.0)
Give GPs access to their patient's hospital records	Give GPs access to their patient's hospital records	1 (0.1)	0 (0.0)
Advocacy organizations facilitate patient-physician communication	Advocacy organizations could mediate patient-physician discussions online, to 'translate' patients' needs to medical language.	1 (0.1)	0 (0.0)
Labs send test results to patients and physicians	Labs send the results of the patient's test to the patient and directly to the physician. The results could also be sent to all of the patient's physicians, if they have multimorbidity.	23 (1.5)	13 (0.8)
Prescriptions sent from physician to pharmacy	Prescriptions (or notification of their extension/renewal) are sent from the physician directly to the patient's pharmacy of choice	17 (1.1)	11 (0.7)
Use a single, shared health record	Establish the use of a single personal health record per patient, accessible online by the patient and all their health care professionals upon authorization by the patient. Physicians will both read this record in consultations, and update it with new information (e.g., notes, lab and examination results, prescriptions) so that all information about the patient is stored in one place. This can enable continuity of care and synchronize care between different specialists. Some participants specifically refer to encouraging French physicians to use the Dossier medical partagé (the virtual, shared personal health record application available to patients in France through the national insurance system).	66 (4.3)	34 (2.2)
**Enhance human intelligence**	**Enhance human intelligence (of the patient or the physician) by using artificial intelligence tools that perform tasks complementary to human cognition.**		
Artificial intelligence tools for diagnosis	Automated tools based on artificial intelligence can support physicians in diagnosis (e.g., in the case of rare diseases which often take a long time to diagnose, partly because of poor physician knowledge).	3 (0.2)	1 (0.1)
Drug interaction checker	Automated tools that check for interactions between medications.	1 (0.1)	0 (0.0)
Symptom-checkers	Use symptom-checker websites.	8 (0.5)	5 (0.3)
Chatbots	Provide patients with chatbots to be used in-between consultations.	2 (0.1)	1 (0.1)
Adjust treatment using artificial intelligence	Use artificial intelligence algorithms to automatically adjust patients' medication dose(s) and send the updated prescription(s) by email.	1 (0.1)	0 (0.0)

© 2021 Oikonomidi T et al. *JAMA Network Open.*

Reminder tools	Provide patients with automated systems that remind them to take their medication, attend consultations or renew their prescription.	5 (0.3)	2 (0.1)
Automated just-in-time adaptive interventions	Adoption of real-time adaptive interventions (e.g., closed loop insulin delivery).	1 (0.1)	0 (0.0)
**Support innovation implementation**	**Support the implementation of the remote parts of blended care or other modifications suggested by participants.**		
Adapt physician billable hours	In the current health insurance system, physicians are compensated on the basis of consultations. This should be adapted to accommodate new forms of care such as asynchronous remote communication with patients.	3 (0.2)	1 (0.1)
Chaperone remote care implementation	Support patients to access and use remote care, such as by creating local telemedicine hubs (i.e., community centres or local medical cabinets equipped with the necessary equipment for remote care, such as monitoring devices the patient can use), or assigning staff to help patients.	8 (0.5)	3 (0.2)
Ensure interoperability between health record software	Refers to making electronic health record software used by physicians compatible with the (national insurance-provided) personal health record, to enable and facilitate its use.	1 (0.1)	0 (0.0)
Fund remote care equipment for physicians	Fund remote care equipment for physicians so they are able to provide remote care services.	1 (0.1)	0 (0.0)
Allocate time for communication between physicians	Similar to scheduled consultations between patient and physician, specific time slots will be allocated for physicians to consult with each other on their common patients.	1 (0.1)	1 (0.1)
Provide patients with physicians' contact information	Patients should be given their physicians' contact information (e.g., phone, email).	3 (0.2)	1 (0.1)
Shared decision-making for remote care modalities	Inform patients about the pros and cons of the remote care modalities introduced in their care.	1 (0.1)	1 (0.1)
Allocate time for remote physician-patient communication	Specific time slots will be allocated for physicians to contact their patients who have initiated asynchronous communication (e.g., by replying to their emails or returning calls).	1 (0.1)	0 (0.0)
Expand functions of personal health records	Expand the functions offered by the patients' personal health records. Participants' suggestions include: the possibility to have teleconsultations via their personal account on the national health record website, visualizations of the evolution of their illness over time, and self-monitoring logs that can be shared with their physician.	4 (0.3)	1 (0.1)

© 2021 Oikonomidi T et al. *JAMA Network Open.*

Reimburse teleconsultations	Provide equal reimbursement for teleconsultations and in-person consultations.	4 (0.3)	1 (0.1)
Adapt teleconsultations for patients with hearing loss	Make teleconsultations accessible for patients with hearing loss, such as by providing simultaneous Sign Language translation.	1 (0.1)	0 (0.0)
Simplify remote monitoring tools	Make remote monitoring tools simple and easy to use for all patients.	1 (0.1)	0 (0.0)
Create thematic email accounts	Health care organizations and physicians can create different email accounts for different patient needs to streamline communications.	1 (0.1)	0 (0.0)
Provide self-monitoring tools	Provide self-monitoring tools that offer data summaries and visualisations, with the possibility to share the monitoring data with physicians.	11 (0.7)	9 (0.6)
Equate remote and in-person prescriptions	Medication prescriptions obtained remotely should have the same validity as those obtain in person (e.g., same duration of validity, ability to prescribe all medications remotely).	3 (0.2)	10 (0.7)
Consultation cap	Set a limit of patients per physician per day (except for emergency services) to allow for more in-depth and longer consultations.	1 (0.1)	0 (0.0)
**Information and education**	**Provide information and education to patients and physicians.**		
Targeted information dissemination	Push-content systems, such as newsletters, will offer information and advice targeted to the patient's illness. This may include news about research on their illness and information on emerging situations that can reassure patients, such as advice for coping with seasonal infectious illnesses for patients with a given chronic illness.	8 (0.5)	5 (0.3)
Online self-management or coaching modules	Coaching and synchronous or asynchronous online courses can provide non-pharmacologic self-management skills to patients, tailored to their illness. Suggested topics include patient education, pain management, exercise and stress management. Suggested formats include videos, live streaming, and apps. The online modules can be stand-alone, or they can be offered to patients after a few in-person sessions.	14 (0.9)	3 (0.2)
Interactive, illness-specific webinars	Refers to patients being able to attend online informational seminars on their illness led by specialists to whom they can also pose questions	5 (0.3)	5 (0.4)
Provide information on treatment options	Provide information about all available treatment options with their pros and cons to patients, to enable decision-making.	3 (0.2)	1 (0.1)
Create reliable websites offering an "illness overview"	Create reliable websites that provide an overview of all necessary information about an illness.	5 (0.3)	1 (0.1)
Specialist directory	Provide patients with a directory of specialists on their illness.	3 (0.2)	1 (0.1)

© 2021 Oikonomidi T et al. *JAMA Network Open.*

Add FAQ section to hospital websites	Add frequently asked questions (FAQ) sections to hospital websites to provide information to patients	1 (0.1)	0 (0.0)
Link GPs to resources via hospital information systems	Link general practitioners to resources via hospital information systems. Resources may include links to associations on specific diseases, etc.	1 (0.1)	0 (0.0)
Patient representative education	Remote education of patient associations or expert patients.	1 (0.1)	0 (0.0)
Physician education	Remote education of physicians.	3 (0.2)	5 (0.3)
**Documentation**	**Provide patients with documents related to their care using remote technologies.**		
Store information on patient's insurance card	Store important information on patients' insurance card. The idea is that as people carry their card with them most of the time, the information would be readily available at emergencies or for patients with memory impairment, and that it provides a practical way of accessing a patient's virtual prescriptions.	7 (0.5)	3 (0.2)
Regular patient briefing	Patients will regularly receive a written summary of their care (e.g., exam results, treatments tested, health events experienced).	2 (0.1)	1 (0.0)
Receive documents by email	Patient receives documents, such as summary reports of consultations, by email (except for prescriptions, lab test results and referral letters, for which separate codes were used).	17 (1.1)	23 (1.5)
**Administrative acts**	**Facilitate administrative tasks.**		
Replace insurance card with face recognition systems	Replace insurance card with face recognition systems.	1 (0.1)	0 (0.0)
Apply for reimbursement online	Facilitate application for reimbursement by giving patients the option to transmit documents online, instead of by post.	4 (0.3)	1 (0.1)
**Change insurance and employment regulations**	**Change regulations regarding the insurance coverage and employment of chronically ill people.**		
Eliminate required referrals	Patients will be able to have a reimbursed consultation with a specialist without a referral letter from their family physician. A referral letter is currently required in France to obtain reimbursement.	3 (0.2)	2 (0.1)
Allow remote work for chronically ill employees	Allow remote work for chronically ill employees.	2 (0.1)	0 (0.0)
**Other**	**Uncategorized codes**		
Wear masks in winter	Wear masks in winter to avoid infectious diseases (e.g., flu).	2 (0.1)	7 (0.5)

[a] Weighted data were obtained after calibration on margins for sex, age, and educational level by using data from a national census describing the French population with chronic illness.

© 2021 Oikonomidi T et al. *JAMA Network Open.*
